# SuperQuant-assisted comparative proteome analysis of glioblastoma subpopulations allows for identification of potential novel therapeutic targets and cell markers

**DOI:** 10.18632/oncotarget.24321

**Published:** 2018-01-25

**Authors:** Thiago Verano-Braga, Vladimir Gorshkov, Sune Munthe, Mia D. Sørensen, Bjarne W. Kristensen, Frank Kjeldsen

**Affiliations:** ^1^ Department of Biochemistry and Molecular Biology, University of Southern Denmark, Odense, Denmark; ^2^ Department of Physiology and Biophysics, Federal University of Minas Gerais, Belo Horizonte, MG, Brazil; ^3^ Department of Pathology, Odense University Hospital, Odense, Denmark; ^4^ Department of Neurosurgery, Odense University Hospital, Odense, Denmark; ^5^ Department of Clinical Research, University of Southern Denmark, Odense, Denmark

**Keywords:** proteomics, glioblastoma, cancer stem cell, cancer proteome, migration

## Abstract

Glioblastoma (GBM) is a highly aggressive brain cancer with poor prognosis and low survival rate. Invasive cancer stem-like cells (CSCs) are responsible for tumor recurrence because they escape current treatments. Our main goal was to study the proteome of three GBM subpopulations to identify key molecules behind GBM cell phenotypes and potential cell markers for migrating cells. We used SuperQuant–an enhanced quantitative proteome approach–to increase proteome coverage. We found 148 proteins differentially regulated in migrating CSCs and 199 proteins differentially regulated in differentiated cells. We used Ingenuity Pathway Analysis (IPA) to predict upstream regulators, downstream effects and canonical pathways associated with regulated proteins. IPA analysis predicted activation of integrin-linked kinase (ILK) signaling, actin cytoskeleton signaling, and lysine demethylase 5B (KDM5B) in CSC migration. Moreover, our data suggested that microRNA-122 (miR-122) is a potential upstream regulator of GBM phenotypes as miR-122 activation was predicted for differentiated cells while its inhibition was predicted for migrating CSCs. Finally, we validated transferrin (TF) and procollagen-lysine 2-oxoglutarate 5-dioxygenase 2 (PLOD2) as potential markers for migrating cells.

## INTRODUCTION

Glioblastoma (GBM), a WHO grade IV glioma, is the most aggressive primary brain cancer [1] with a median survival rate of only 15 months [2]. Surgical resection combined with radiotherapy and chemotherapy is the standard treatment for GBM [2]. However, tumor recurrence is inevitable because GBM cells can invade surrounding tissues thus preventing full removal of tumor cells by resection. Moreover, subpopulations of cancer stem-like cells (CSCs), which are resistant to radiotherapy and chemotherapy, are present in tumor environment [3, 4]. CSCs self-renew and recapitulate the original tumor upon cerebral implantation in mice [5]. Evidences suggest that CSCs have developed a number of molecular mechanisms to escape and survive treatments. For example, radiation resistance seems to be due to increased activity of the DNA damage response machinery in CSCs [3]. Radiotherapy with concomitant temozolomide (TMZ) has increased the survival rate by several months [2], however, recurrence is still observed after treatment.

Understanding the regulatory system of CSCs is critical to design next-generation drugs to ultimately imbalance CSC homeostasis. Regulation of CSCs in gliomas involves extrinsic (microenvironment, niche factors and host immune system) and intrinsic (genetic, epigenetic, and metabolic) factors [6]. For example, CSCs maintain an undifferentiated state by aberrant activation of common signaling pathways such as Notch, NF-κB, BMP, Wnt, and PI3K-AKT signaling [6].

A number of biomarkers are available for glioma cells, such as mutation of isocitrate dehydrogenase 1 (mIDH1) [7, 8], methylation of MGMT gene (DNA repair enzyme O^6^-methylguanine-DNA methyltransferase) [9], as well as standard stem cell markers including, but not restricted to, CD133, Musashi-1, Bmi-1, Sox-2 and Nestin [10, 11]. Some CSCs are able to migrate from the tumor core, representing a significant challenge for treatment as they are left behind after tumor resection [9, 12]. Although standard stem cell markers can be used to identify migrating CSCs, these markers cannot be used to select the migrating phenotype from other CSCs.

Gene expression analysis is a valuable method in cancer research. However, gene counts do not necessarily correlate with protein abundances in cells due to mRNA regulation and protein turnover. During the past decade, mass spectrometry (MS)-based proteomics has become the leading technology to study biological systems. Our group developed the Complementary Finder software. This tool uses high-confidence complementary fragment ions to derive individual and unique peptide parent masses, deconvoluting mixture spectra and increasing proteome coverage [13]. More recently, we implemented the SuperQuant method to infer quantitative information to the output data of Complementary Finder using MS^1^ spectrum-based quantitation. On average, SuperQuant allows the identification and quantitation of 10% more proteins per study enabling a more insightful proteome interpretation [14].

Given the poor prognosis of GBM, the identification of specific biomarkers and therapeutic targets for this disease are of imminent importance to improve the early diagnosis and treatment, including the development of personalized medicine [15]. In this study, our goals were to identify key regulators and cell markers from different phenotypes of GBM cells, focusing on the migrating CSC phenotype. We used SuperQuant to assess deeper into the GBM proteome of CSC spheroids, migrating CSCs and differentiated GBM cells. We identified and quantified the protein abundances of more than 2,817 proteins, of which 304 proteins were found differentially regulated. Among regulated proteins, we identified potential therapeutic targets and cell markers that could serve as subjects for further investigation.

## RESULTS AND DISCUSSION

### Experimental design used to study proteins involved in the GBM migration and differentiation

**Figure 1:**
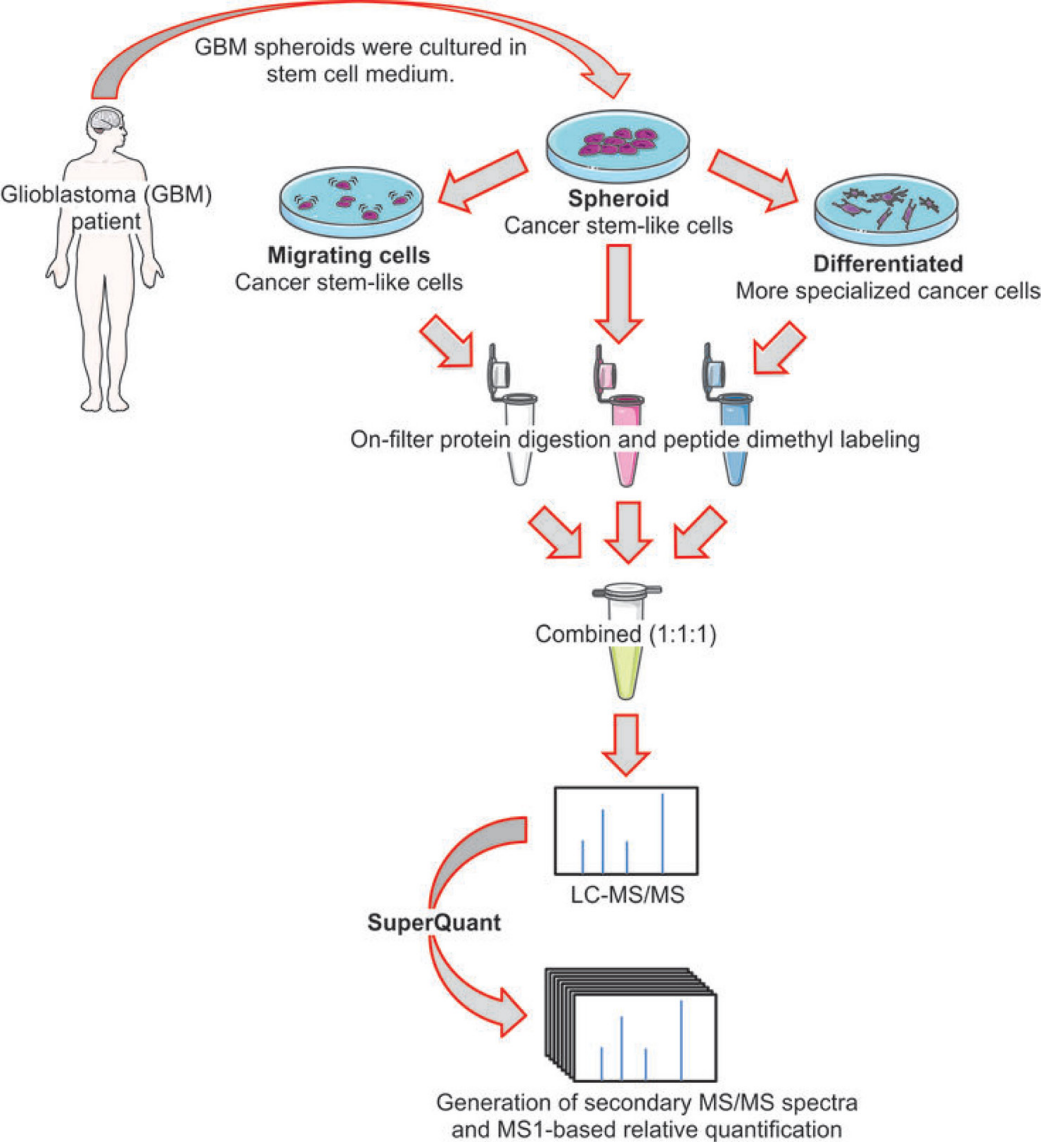
Experimental setup Glioblastoma stem cell-like spheroids were cultured using the serum-free medium to maintain the stem-like phenotype. Migrating cancer stem-like cells (CSCs) were selected from CSC spheroids using the migration assay reported previously and represented in Supplementary Figure 1. Differentiated cells were obtained by culturing CSC spheroids in medium containing 10% (w/v) fetal calf serum. After cell lysis and on-filter protein digestion, peptides were labeled with dimethyl labeling. SuperQuant was used to increase quantitative proteome coverage.

We used the proteomics pipeline represented in Figure 1 to compare the proteome of three GBM cell phenotypes. Tissue fragments from a grade IV GBM patient subjected to brain tumor resection were obtained in a study published previously [16]. Tissue fragments were dissociated to the single cell level and thereafter they formed spheroids in serum free medium. We decided to grow the tumor cells as organotypic tumor spheroids (OTS) because 3D culture preserves important features of the original tumor tissue, including cell-to-cell interactions, thus representing a more *in vivo*-like model [17, 18]. Migrating and differentiated cells were obtained from CSC spheroids. To select CSCs with migrating phenotype, we used the migration assay reported by Munthe *et al.* [19] and represented in the supporting information (Supplementary Figure 1). The migration assay was conducted with stem cell medium to maintain cells undifferentiated. On the other hand, to produce the differentiated cells we cultured the CSC spheroids with medium containing 10% (w/v) fetal calf serum (FCS) (Figure 1).

*In vitro* experiments performed with neural stem cells showed that these cells expand and form neurospheres when grown under serum-free conditions [20]. Lee *et al.* showed that GBM cells cultured under serum-free conditions more closely resembled primary GBMs and preserved CSC features [21]. Therefore serum-free medium more accurately reflect the cell environment in brain tissue compared to serum-containing medium for culturing CSCs.

Recently we reported a new post-acquisition method (named SuperQuant) that allows increased quantitative proteome depth by extracting co-isolated/co-fragmented peptides from MS/MS output files that are neglected in a normal shotgun proteomic analysis [14]. SuperQuant processing led to the identification and quantitation of 2,817 protein groups, representing an increment of 7% compared to unprocessed data (Supplementary Figure 2, supporting information). This improvement is comparable with our previous study reporting approximately 10% increase of quantified proteins using HeLa cells [14]. To evaluate quantitation reproducibility of our method, we calculated the coefficient of variance (CV) between replicates for each protein. The CVs distributions are presented in Supplementary Figure 3 (supporting information). The average CV between replicate experiments was 3.5%.

### The proteome of CSC spheroids and migrating CSCs are more similar than the proteome from differentiated cells

Since migrating CSCs and differentiated cells were generated from CSC spheroids, we used the dataset of CSC spheroids as reference (denominator) to obtain protein ratios. As consequence, upregulation of a given protein from the migrating CSCs dataset means that this protein is statistically more abundant in migrating CSCs than in CSC spheroids. Likewise, down-regulation of a given protein from differentiated cells means that this protein is differentially more abundant in CSC spheroids than in differentiated cells.

Out of 2,817 proteins detected and quantified in this study, 199 and 148 were differentially regulated in the differentiated and migrating CSCs datasets, respectively (Supplementary Table 1). Comparing the regulated proteins from both datasets, we observed that only 43 proteins (14%) were identified in both datasets (Supplementary Figure 4), indicating that the differential proteomes from migrating CSCs and differentiated cells are rather different.

**Figure 2:**
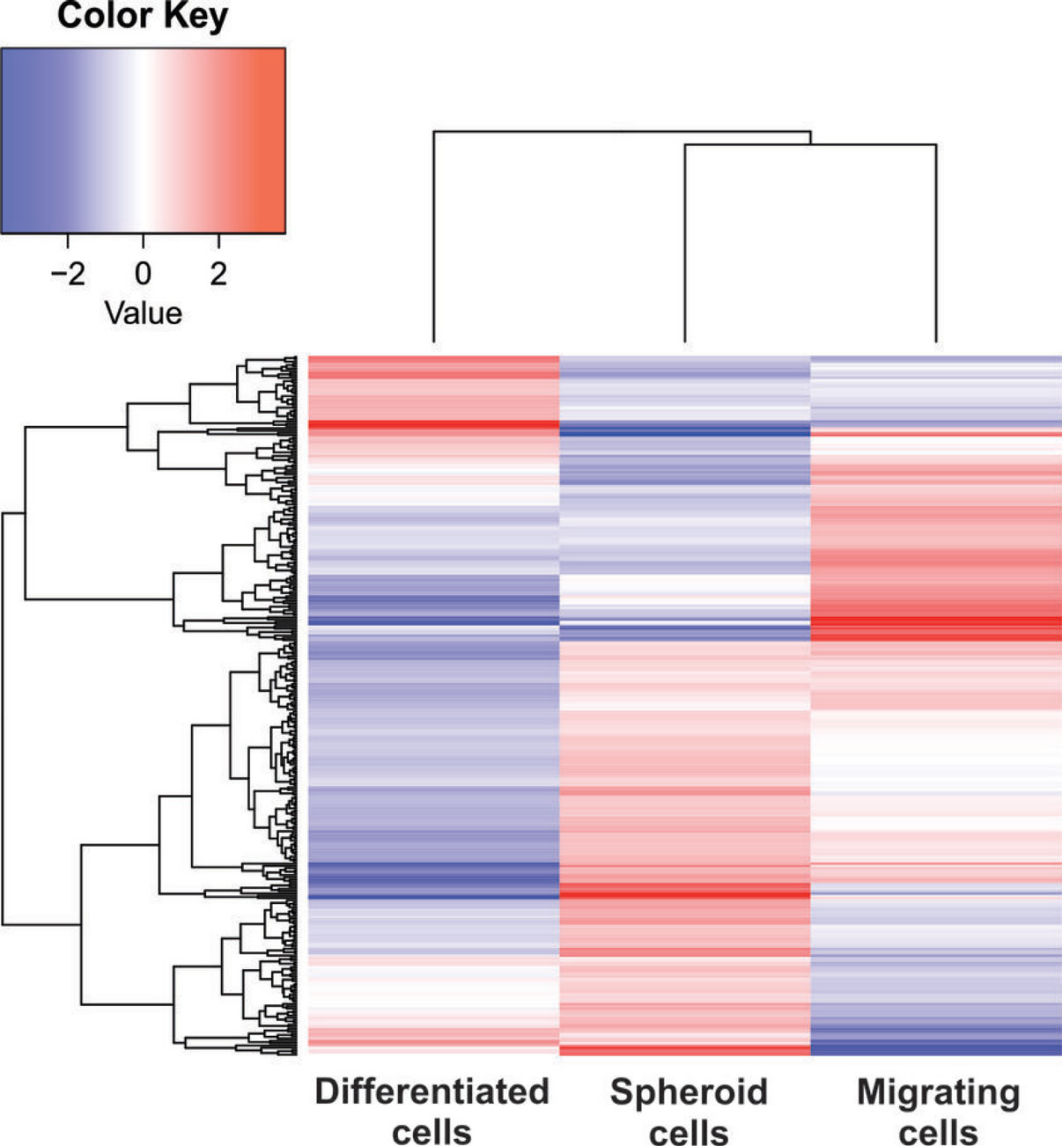
Hierarchical clustering analysis Log-transformed protein abundances of differentially regulated proteins (*q*-value < 0.05) from CSC spheroids, migrating CSCs, and differentiated cells.

Hierarchical clustering analysis shows that the proteome of CSC spheroids and migrating CSCs are more similar than the proteome of differentiated cells (Figure 2). Although the similarity is only marginal in relation to the proteome of the differentiated cells, this result is likely to reflect the stem-like phenotype of spheroids and migrating cells [19]. However, we would like to point out that even these CSCs have clusters containing proteins with opposing abundance profiles indicating genes with distinct regulations.

### Pathway analysis of migrating CSCs and differentiated cells

Activation z-score in IPA was used to predict modulation patterns of the top-scoring canonical pathways. This algorithm uses the causal network to predict activation or inhibition of given pathways based on experimental data and expected expression levels of signaling effectors from a curated biological database (Ingenuity Knowledge Base) [22].

**Figure 3:**
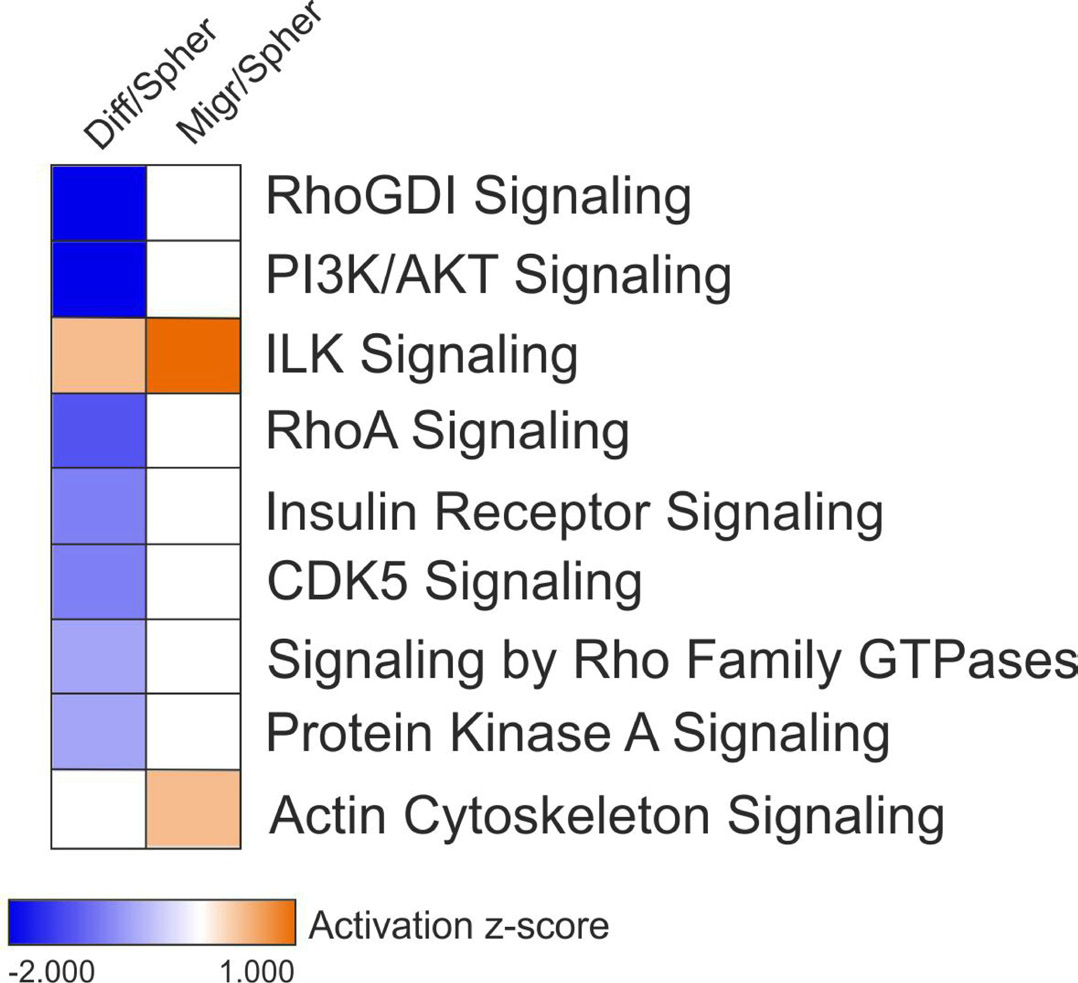
Modulation of canonical signaling pathways in migrating CSCs and differentiated cells Activation z-score for top-scoring canonical pathways from differentiated cells (left column) and migrating CSCs (right column). Positive values for activation z-score are in orange and denote activation. Negative values for activation z-score are in blue and indicate inhibition. Z-scores equal or very close to zero are in white and indicate no modulation. Not-defined z-score values are also in white.

As shown in Figure 3, IPA predicted opposing modulations for all top-scoring canonical pathways from migrating CSCs and differentiated cells, except for the integrin-linked kinase (ILK) signaling, predicted to be activated in both datasets. ILK connects integrin receptors with the actin cytoskeleton to regulate many cellular processes including proliferation, angiogenesis, survival, differentiation, migration, and invasion [23]. The highest activation z-score was calculated for migrating cells suggesting that ILK signaling is more active in migrating CSCs. Data suggest that activation of ILK signaling is important for migration of CSCs and differentiation. Since GBM recurrence after treatment is thought to be due to CSCs [6], our data suggest that inhibition of ILK signaling can be a potential treatment for GBM. In fact, a recent study has shown that p53-wildtype GBM cells were more susceptible to radiotherapy after the knockdown of ILK and its signaling partners PINCH1 and ILKAP [24].

Activation of actin cytoskeleton signaling was predicted only for migrating CSCs (Figure 3). Actin cytoskeleton reorganization is a key process in migration of GBM cells. Migrating cells produce actin-rich protrusions of the plasma membrane, named invadopodia, which interact with the extracellular matrix (ECM) inducing its proteolysis and remodeling. The presence of invadopodia is usually associated with poor prognosis [25, 26].

As shown in Figure 3, IPA predicted inhibition of RhoGDI, PI3K/AKT, RhoA, insulin receptor, CDK5, Rho family GTPases, and protein kinase A (PKA) signaling pathways for differentiated cells. This data suggest that the inhibition of these signaling pathways is important for GBM differentiation. Regarding PI3K/AKT signaling, its activation is a key mechanism in glioma progression to higher-grade tumors. Increased activity of PI3K/AKT in CSCs induces resistance by enhancing expelling of drugs via ABC transporters [27]. Moreover, CSC is maintained undifferentiated by aberrant activation of PI3K/AKT [6].

### Prediction of upstream regulators for migrating CSC and differentiated cell proteomes

**Figure 4:**
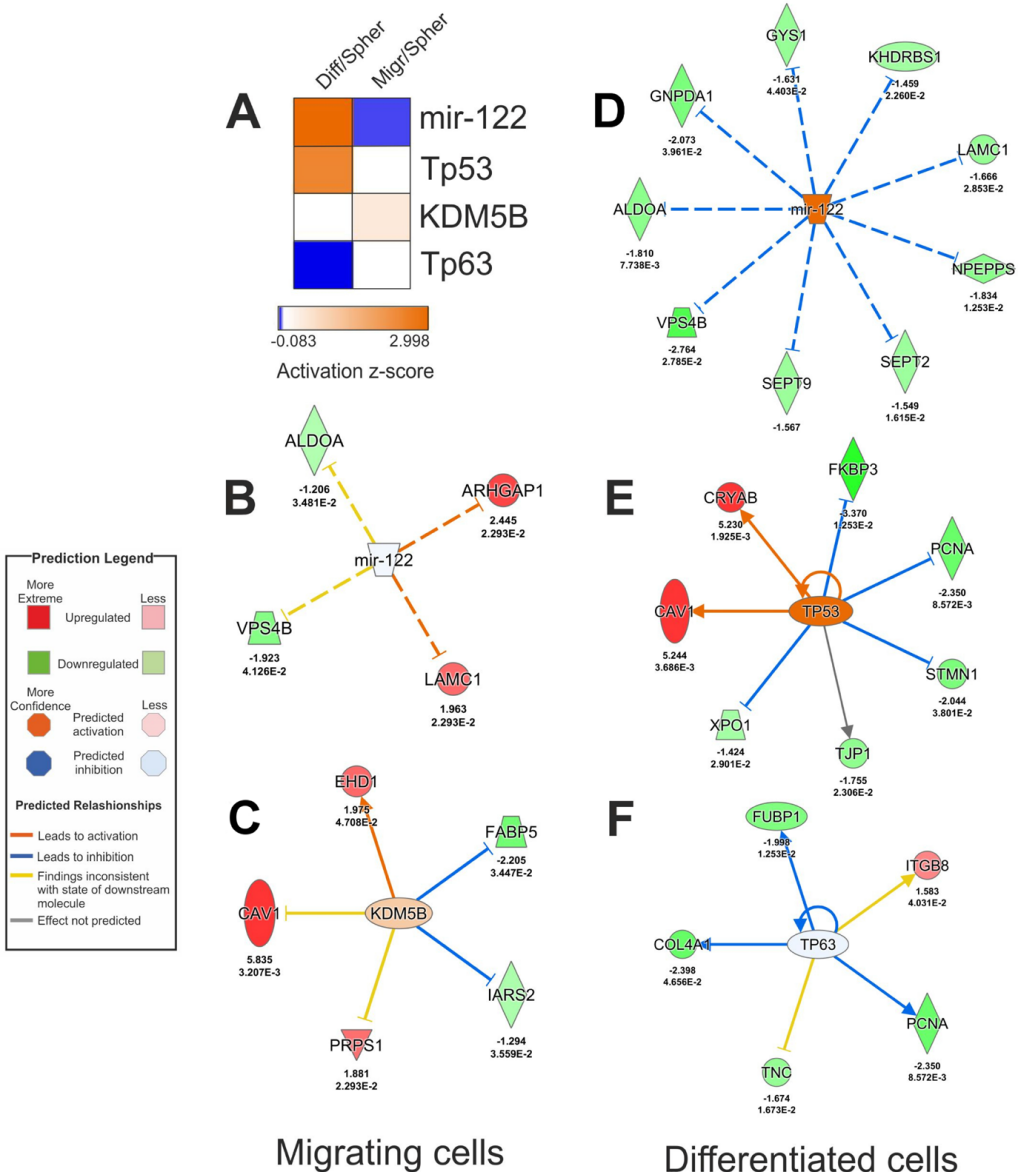
Potential upstream regulators of migrating CSC and differentiated cancer cell proteomes (**A**) Heat map with activation z-scores of predicted upstream regulators. (**B**–**F**) Causal connections between predicted upstream regulators and their targets from migrating (B–C) and differentiated (D–F) proteomes. Experimental log-transformed ratios and *q*-values are represented below each protein associated with upstream regulators. More information related to upstream regulators and their associated proteins from differentiated and migrating proteomes are provided in Table 1 and Table 2, respectively.

Figure 4A shows the predicted upstream regulators. For the differentiated cells, IPA predicted activation of microRNA 122 (miR-122) (z-score = 2.998) and tumor antigen p53 (Tp53) (z-score = 2.425), and inhibition of tumor antigen p63 (Tp63) (z-score = −0.083). For migrating CSCs, prediction outcomes were activation of lysine demethylase 5B (KDM5B) (z-score = 0.447) and inhibition of miR-122 (z-score = −0.061). Figure 4B–4F represents causal associations between the predicted upstream regulators and their protein targets (experimental data). Annotations retrieved from IPA are provided in Table 1 (differentiated dataset) and Table 2 (migrating dataset).

**Table 1:** Differentiated proteome: predicted upstream regulators and downstream effectors

Symbol	ID	Name	Location	Type(s)
miR-122^*^	—	microRNA 122	Cytoplasm	microRNA
ALDOA	P04075	aldolase, fructose-bisphosphate A	Cytoplasm	Enzyme
GNPDA1	P46926	glucosamine-6-phosphate deaminase 1	Cytoplasm	Enzyme
GYS1	P13807	glycogen synthase 1	Cytoplasm	Enzyme
KHDRBS1	Q07666	KH RNA binding domain containing, signal transduction associated 1	Nucleus	transcription regulator
LAMC1	P11047	laminin subunit gamma 1	Extracellular Space	Other
NPEPPS	P55786	aminopeptidase puromycin sensitive	Cytoplasm	peptidase
SEPT2	Q15019	septin 2	Cytoplasm	Enzyme
SEPT9	Q9UHD8	septin 9	Cytoplasm	Enzyme
VPS4B	O75351	vacuolar protein sorting 4 homolog B	Cytoplasm	transporter
Tp53^*^	—	tumor protein p53	Nucleus	transcription regulator
CAV1	Q03135	caveolin 1	Plasma Membrane	transmembrane receptor
CRYAB	P02511	crystallin alpha B	Nucleus	Other
FKBP3	Q00688	FK506 binding protein 3	Nucleus	Enzyme
PCNA	P12004	proliferating cell nuclear antigen	Nucleus	Enzyme
STMN1	P16949	stathmin 1	Cytoplasm	Other
TJP1	Q07157	tight junction protein 1	Plasma Membrane	Other
XPO1	O14980	exportin 1	Nucleus	transporter
Tp63^*^	—	tumor protein p63	Nucleus	transcription regulator
COL4A1	P02462	collagen type IV alpha 1 chain	Extracellular Space	Other
FUBP1	Q96AE4	far upstream element binding protein 1	Nucleus	transcription regulator
ITGB8	P26012	integrin subunit beta 8	Plasma Membrane	Other
PCNA	P12004	proliferating cell nuclear antigen	Nucleus	enzyme
TNC	P24821	tenascin C	Extracellular Space	Other

Molecule annotation was retrieved from IPA. (^*^) indicates the predicted upstream regulator.

**Table 2:** Migrating proteome: predicted upstream regulators and downstream effectors

Symbol	ID	Name	Location	Type(s)
miR-122^*^	—	microRNA 122	Cytoplasm	microRNA
ALDOA	P04075	aldolase, fructose-bisphosphate A	Cytoplasm	enzyme
ARHGAP1	Q07960	Rho GTPase activating protein 1	Cytoplasm	other
LAMC1	P11047	laminin subunit gamma 1	Extracellular Space	other
VPS4B	O75351	vacuolar protein sorting 4 homolog B	Cytoplasm	transporter
KDM5B^*^	—	lysine demethylase 5B	Nucleus	transcription regulator
CAV1	Q03135	caveolin 1	Plasma Membrane	transmembrane receptor
EHD1	Q9H4M9	EH domain containing 1	Cytoplasm	other
FABP5	Q01469	fatty acid binding protein 5	Cytoplasm	transporter
IARS2	Q9NSE4	isoleucyl-tRNA synthetase 2, mitochondrial	Cytoplasm	enzyme
PRPS1	P60891	phosphoribosyl pyrophosphate synthetase 1	Cytoplasm	kinase

Molecule annotation was retrieved from IPA. (^*^) indicates the predicted upstream regulator.

MicroRNAs (miRNAs) are noncoding RNA that suppress expression of target genes by degrading correspondent mRNAs or inhibiting their translation process [28]. Deregulation of some miRNAs has been reported in brain cancer including miR-124a, a miRNA potentially involved in GBM invasion [29]. IPA predicted miR-122 inhibition for migrating CSCs and miR-122 activation for GBM differentiation (Figure 4A). Thus, our data suggest that miR-122 is a potential regulator of GBM fate. By functioning as an “ON/OFF button”, miR-122 seems to control GBM migration and differentiation. IPA analysis correlated miR-122 inhibition in migrating CSCs with the experimentally observed upregulation of ARHGAP1 and LAMC1 (Figure 4B). Although, the relationship between ARHGAP1 and GBM migration is yet to be determined, LAMC1 is indeed involved in GBM migration [29]. IPA indicated inconsistent findings for ALDOA and VPS4B. Based on the Ingenuity Knowledge Base [22], inhibition of miR-122 correlates with upregulation of ALDOA and VPS4B, but we detected downregulation of these proteins in migrating CSCs (Figure 4B), which suggests involvement of other upstream regulators than miR-122 regulating the migrating CSCs proteome. As shown in Figure 4D, activation of miR-122 in differentiated correlated with downregulation of ALDOA, GNPDA1, GYS1, KHDRBS1, LAMC1, NPEPPS, SEPT2, SEPT9, and VPS4B indicating that these downregulation events are potentially related to GBM differentiation. Similarly, activation of Tp53 (Figure 4E) and inhibition of Tp63 (Figure 4F) may also have a potential role in GBM cell differentiation.

MiR-122 is a liver-specific miRNA [30]. However, this miRNA seems to be less tissue-specific in cancer as miR-122 deregulation has been associated with colorectal cancer [31, 32], breast cancer [33], and gliomas [34], although miR-122 plays a role in hepatocellular carcinoma [35].

IPA predicted activation of KDM5B for migrating CSCs highlighting its potential role in promoting CSC migration. KDM5 activation was consistent with EHD1 upregulation (Figure 4C). Interestingly, EHD1 has been reported as a potential inducer of cell migration and metastasis in non-small cell lung cancer [36]. A previous study showed high levels of KDM5B in glioma, inducing tumor growth via p21 downregulation [37]. Our data suggest a new role for KDM5B as a regulator of CSCs migration in GBM. In line with this observation, increased expression of KDM5B has been reported in metastatic breast cancer cells highlighting its potential involvement in cancer cell migration [38].

**Figure 5:**
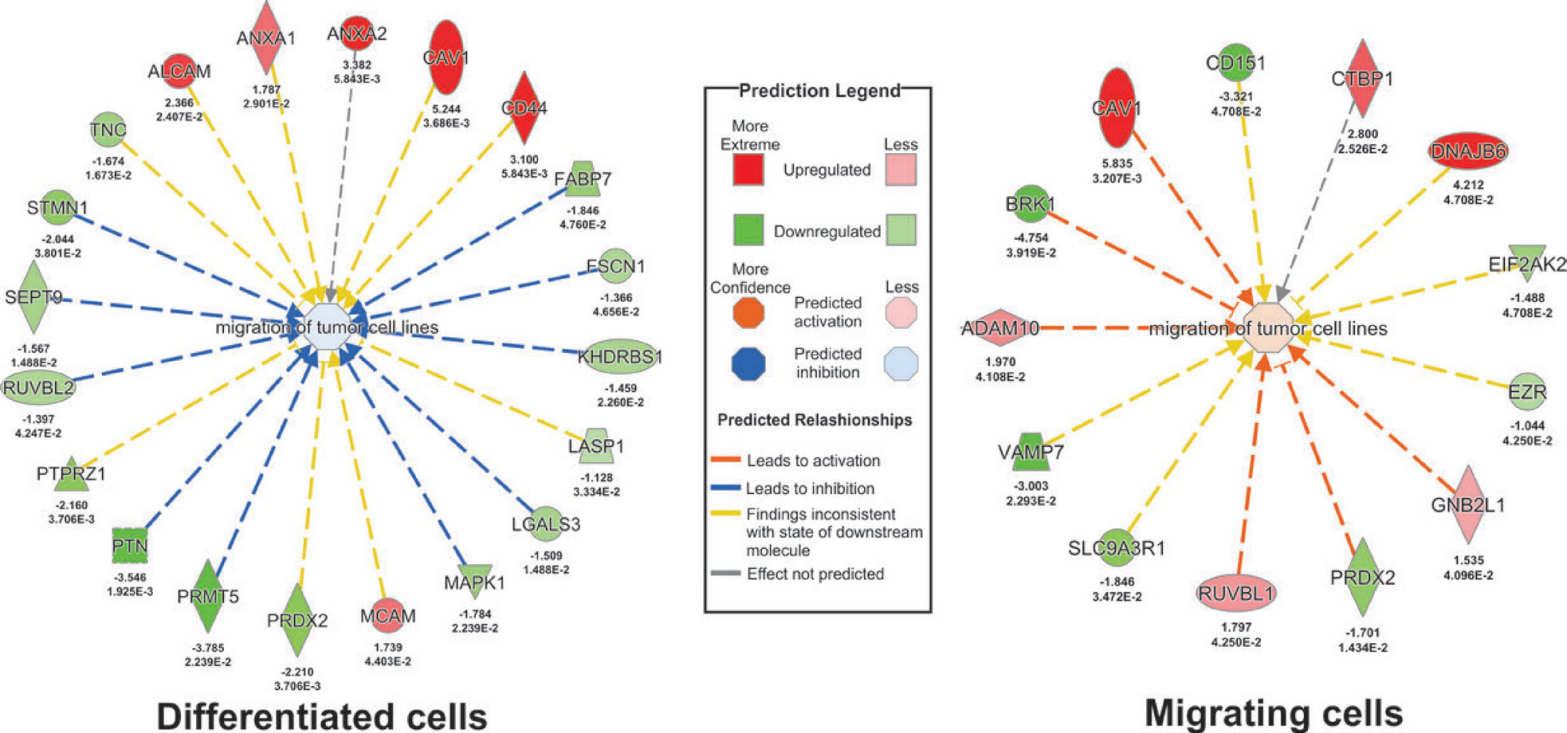
Profiling the proteins potentially involved in the migration of GBM cells Regulated proteins potentially involved in the enriched downstream effect “migration of tumor cell lines” are shown.

After shedding light on potential upstream proteome regulators from migrating CSC and differentiated cells, we used the IPA downstream effect analysis. The downstream effect “migration of tumor cell lines” was found significantly regulated in the migrating CSCs (*p*-value = 0.007) and differentiated cells (*p*-value = 0.0003), which was predicted to be activated in migrating CSCs (z-score = 0.04) and inhibited in differentiated cells (z-score = −0.158) (Figure 5). Upregulation of CAV1, GNB2L1, RUVBL1, and ADAM10 as well as downregulation of PRDX2 were consistent with activation of “migration of tumor cell lines” (right panel, Figure 5). Although IPA analysis did not predict upregulation of CTBP1 as an activator of migration of cancer cells, a recent report showed that silencing of CTBP1 suppressed migration of human glioma cells, indicating the potential role of CTBP1 in GBM invasiveness [39]. Inhibition of “migration of tumor cell lines” was consistent with downregulation of FABP7, FSCN1, KHDRBS1, LGALS3, MAPK1, PRMT5, PTN, RUVBL2, SEPT9, and STMN1 (left panel, Figure 5). IPA reported “inconsistent findings with the state of downstream molecule” (yellow arrows) for both datasets indicating that IPA predicted opposing protein abundances. Importantly, we included “all human tissues, cells and cell lines” for this particular analysis. Thus, the term “migration of tumor cell lines” also includes other cancer cell lines than glial cells, which could explain the observed inconsistent findings as a given protein may favor migration of one type of cancer cell line and inhibition of other.

### Profiling potential phenotype-specific markers

Clinicians and researchers face a great challenge dealing with GBM due to its heterogeneity since cancer cells with different phenotypes populate the tumor mass and surrounding areas. Since CSCs are regarded as key players in tumor recurrence, it is important to identify this phenotype. Stem cell markers such as CD133 [10], Musashi-1 [10], Bmi-1 [10], Sox-2 [10], Nestin [11], and Glut-3 [40] (to review other CSCs markers, see [6]) can be used to identify CSCs as well. However, we lack markers to identify different CSCs phenotypes as, for example, migrating and static CSCs [9, 19].

**Figure 6:**
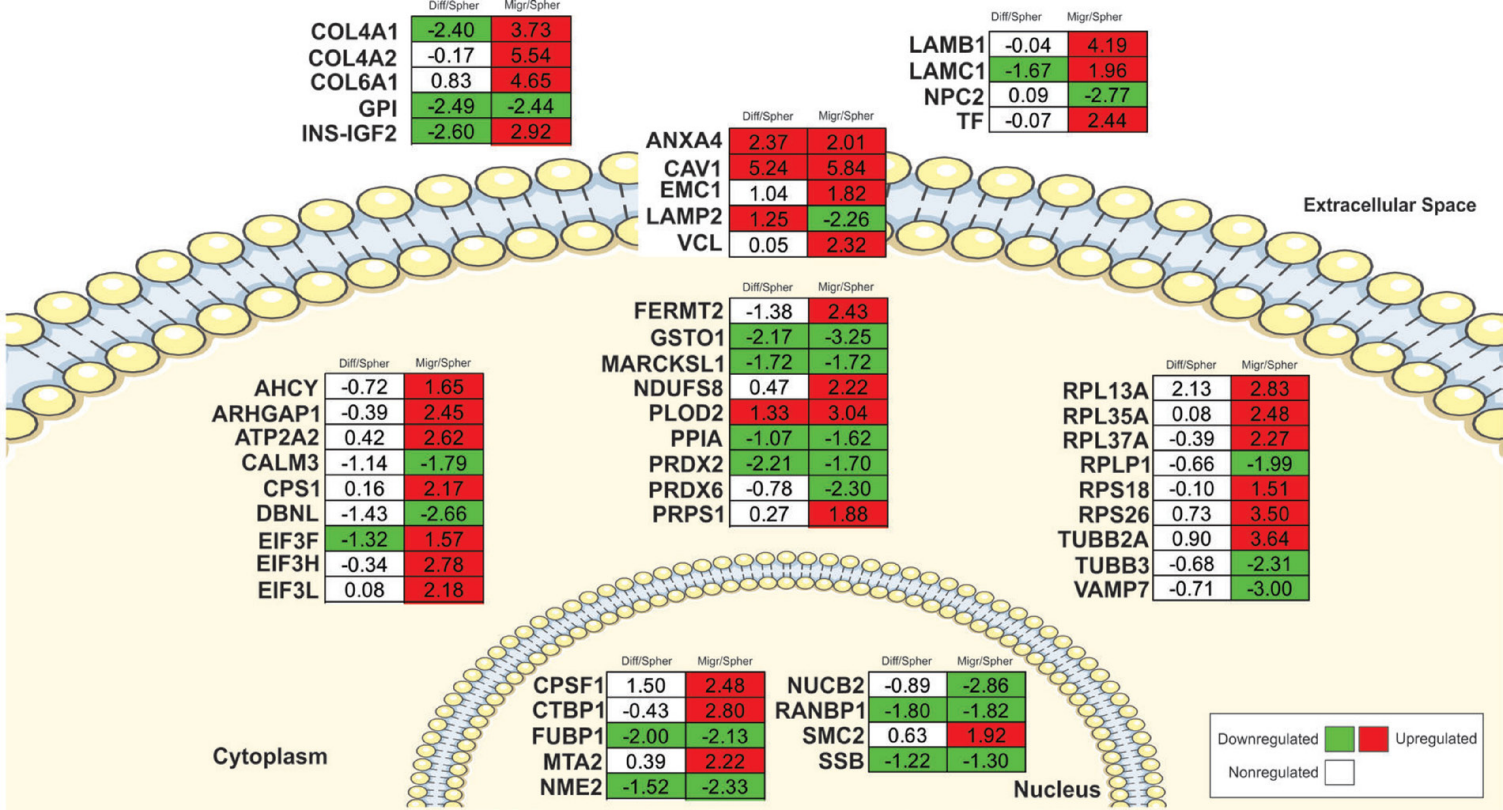
Potential markers for migrating CSCs The top-50 most confident regulated proteins (lowest *q*-values) from migrating CSCs are plotted. Numbers represent the log-transformed ratios. Numbers in the left column are from the differentiated dataset while numbers in the right column are from the migrating CSC dataset. Downregulation (*q*-value < 0.05) = green; upregulation (*q*-value < 0.05) = red; non-regulation (*q*-value > 0.05) = white. Cell localizations were retrieved from Ingenuity Pathway Analysis (IPA). Diff, differentiated; Migr, migrating CSCs; Spher, spheroid CSCs.

To select potential markers for the migrating CSC phenotype, we focused the analysis on the top-50 proteins with lowest *q*-values from the migrating dataset. Figure 6 represents the selected top-50 proteins including their cellular localization (i.e., extracellular, plasma membrane, and intracellular). Based on their abundance profile, proteins were divided into 32 upregulated and 18 downregulated, representing proteins more abundant in migrating CSCs or more abundant in CSC spheroids, respectively. To select potential markers for migrating CSCs, we focused our analysis on the 32-upregulated proteins (Figure 6). We selected candidates for further validation based on antibody availability and novelty (not previously used as GBM migration marker). By using these criteria, we selected VCL and TF for further analysis. Although we observed upregulation of PLOD2 in the differentiated dataset as well, we selected PLOD2 for further validation because this enzyme has already been highlighted as a potential migration marker in other cancers, such as bladder cancer [41] and renal cell carcinoma [42]. Although PTN was not found differentially regulated in the migrating dataset (*q*-value = 0.485; Supplementary Table 1), we also selected this protein for validation because of reports indicating its role in glioma cell migration [43, 44].

We performed *in situ* immunohistochemistry using the same–but non-cultured–tumor tissue from which we obtained the spheroids to validate the selected proteins identified in our SuperQuant-assisted proteomic approach. A total of 14 areas (7 tumor core and 7 tumor periphery) were evaluated by manual tumor cell counting.

**Figure 7:**
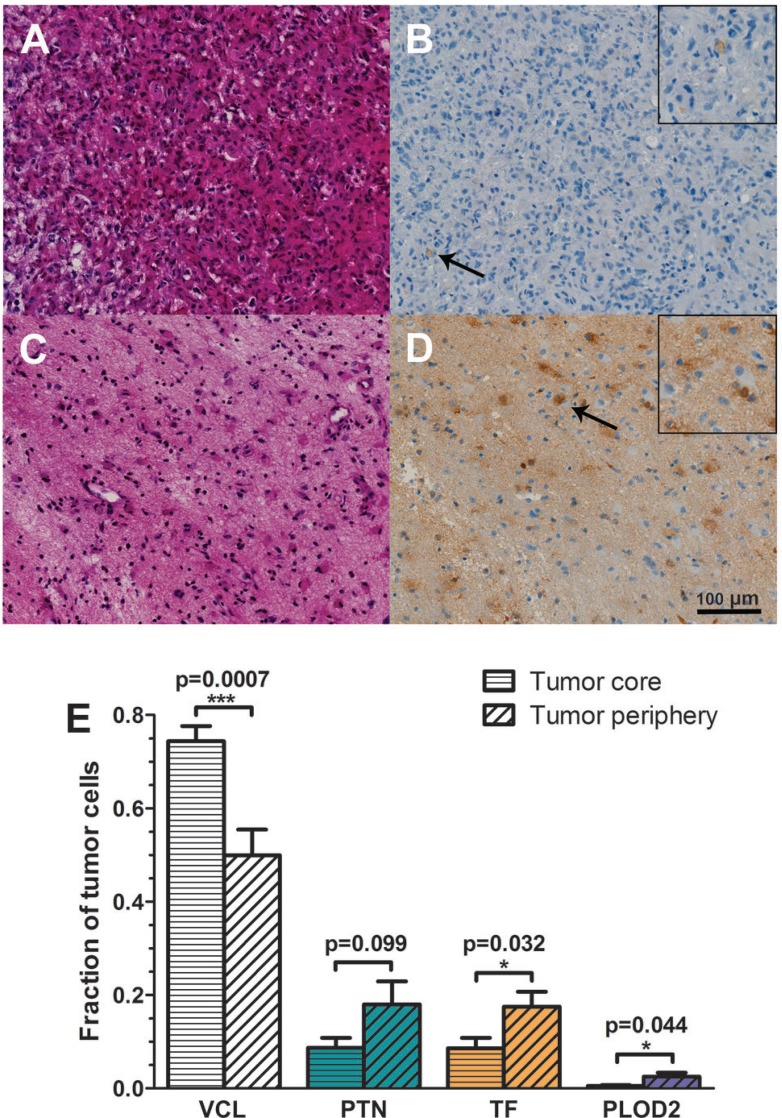
Validation of some potential GBM population-specific marker candidates using immunohistochemical staining of histological sections Transferrin (TF) expression was monitored in tumor core (**A** and **B**) and periphery (**C** and **D**). In A and C, tissue was stained with haematoxylin-eosin (HE) to define tumor core and tumor periphery. In B and D, tissue was immunohistochemically stained using anti-TF antibody. Few tumor cells expressed transferrin in the tumor core (B) while higher abundance of TF was found in the tumor periphery (D). Arrows highlight cells expressing TF and these areas are magnified in the inserts. (**E**) Fractions of cells positive for vinculin (VCL), pleiotrophin (PTN), transferrin (TF) and procollagen-lysine, 2-oxoglutarate 5-dioxygenase 2 (PLOD2) in tumor core and periphery.

Figure 7A–7D contains representative pictures of tissue immunostaining of TF expression in tumor core and periphery. We provided in the supporting information (Supplementary Figure 5) the immunostaining pictures of VCL, PTN, and PLOD2.

In line with the SuperQuant-assisted proteomics data, both TF (*p*-value = 0.032) and PLOD2 (*p*-value = 0.044) were significantly more abundant in the tumor periphery than the tumor core (Figure 7E). TF is involved in the main iron uptake route in animals, and hence one of the most abundant proteins in plasma. Although TF is primarily synthesized in the liver, human brain (mainly oligodendrocytes) synthesizes TF as well [45, 46]. We found a strong upregulation of TF in migrating CSCs (Figure 6), which could be validated by immunohistochemistry (Figure 7D and 7E). Schonberg *et al.* [47] have recently shown that the TF gene is highly expressed in GBM CSCs while its expression is lower in normal neurons and glial progenitors. Furthermore, the authors observed a trend towards worse survival in patients with high levels of TF.

The elevation of PLOD2 in migrating cells is well in-line with other reports showing that PLOD2 is involved in cancer cells migration. Eisinger-Mathason *et al.* [48] showed that hypoxia in the tumor microenvironment and the expression of hypoxia-inducible factor-1α (HIF-1α) led to upregulation of PLOD2 in sarcoma. In addition, Chang *et al.* [49] observed high expression level of PLOD2 in invasive ductal carcinomas of breast cancer. PLOD2 increases collagen stability by hydroxylation of lysine residues in procollagen [50] leading to crosslinked collagen fibers that induce ECM stiffness favoring cell migration, invasion, and metastasis [51]. Furthermore, high expression levels of PLOD2 correlated with poor prognosis in patients with hepatocellular carcinoma [52] and breast carcinoma [49]. Based on mRNA sequencing obtained from laser-capture microdissection of whole tumors, Dong *et al.* [53] suggested PLOD2 as a prognostic biomarker in patients with GBM. Authors observed differential upregulation of PLOD2 in perinecrotic palisading cells–thought to be the most aggressive tumor cell type–compared to non-palisading cells. Moreover, a lower survival rate was associated with patients expressing higher levels of PLOD2 [53]. Our data add an additional level of information to this study as we detected PLOD2 predominantly upregulated in migrating CSCs and to a lesser extent in differentiated cells. Data indicate both TF and PLOD2 as potential markers for migrating GBM cells. However, our results indicated that PLOD2 should not be used as a CSC marker since PLOD2 was also detected upregulated in the differentiated cells (Figure 6 and Supplementary Table 1).

VCL abundance was significantly lower in tumor periphery compared to tumor core. According to the immunostaining, VCL was approximately 33% less abundant in peripheral cells than central cells (Figure 7E). This stands in contrast to our proteomic data showing > 4-fold upregulation of VCL in migrating CSCs compared to CSC spheroids (Figure 6 and supporting information, Supplementary Table 1). Higher VCL-staining in tumor core may be a reflection of the recruitment of VCL-positive microglial cells towards the tumor site [54]. Ubiquitously expressed, VCL is involved in cell-cell interaction and cell-matrix adhesion [55]. According to literature, there is diverging information of the role of VCL in cancer biology. VCL has been reported as tumor suppressor, illustrated by VCL downregulation in highly metastatic colorectal cancer [56], breast cancer [57], squamous carcinoma [58], and rhabdomyosarcoma [59]. In contrast, VCL has been found significantly upregulated in pancreatic cancer tissue [60] as well as in human basal and squamous cell tumors [58]. The diverging results suggest that VCL behavior in cancer cells is potentially tissue-specific.

Secreted PTN is a heparin-binding growth factor [61] and has an important role during brain development reaching its maximum expression level around birth (see [62] for review). PTN abundance was not found significantly regulated in the migrating CSCs dataset (Supplementary Table 1). Likewise, no significant change in PTN abundance was detected in tumor tissue although a trend towards increased levels was observed in the periphery (Figure 7E). These data stand in contrast to the literature reporting that PTN induces migration of some GBM cell lines via the receptor tyrosine phosphatase PTPRZ1 [43, 44]. We believe that tumor heterogeneity and/or the subtype of GBM used in the studies [63] may be responsible for such diverging results.

## CONCLUSIONS

Subjecting GBM subpopulations to a comprehensive quantitative proteome analysis revealed 304 differentially regulated proteins. These proteins represent potential biomarker that may assist researchers and clinicians to accurately select and identify different GBM cell phenotypes, holding potential as next-generation brain cancer diagnostics and therapeutics. Our study indicated miR-122, KDM5B, Tp53, and Tp63 as potential regulators of GBM cell phenotypes.

## MATERIALS AND METHODS

### Reagents

If not explicitly stated, all common solutions and reagents were purchased from Sigma-Aldrich and were of HPLC gradient grade or proteomics grade (when applicable).

### Patient tissue and cell culture

Organotypic tumor spheroids (OTS) were obtained from fresh tumor tissue from a WHO grade IV GBM patient (referred to as T78) who underwent surgery in 2009 as previously described [16]. Cells were cultured as free-floating spheroids in serum-free neural stem cell medium [16] at 36° C in a humidified incubator with 5% CO_2_. We established this protocol to obtain CSCs in our laboratory [17] in accordance with the Regional Scientific Ethical Committee (approval number S-VF-20040102). These cells have the ability to form new spheroids at clonal density. They have a karyotype typical of GBMs, and the ability to form highly invasive tumors upon orthotopic xenografting. Moreover, they differentiate into cells expressing neuronal, astrocytic and oligodendrocyte markers upon culturing in serum-containing medium. The GBM spheroids from T78 have a hypermethylated O6-methylguanine-DNA methyltransferase (MGMT) promoter region and were derived from wildtype isocitrate dehydrogenase 1 (wtIDH1) tumors [16, 64].

### Migration assay

Geltrex (Gibco) and serum-free medium were mixed (1 + 49), and 1400 μL was added to each well in 12-well plates. Coated plates were incubated overnight at 36° C, and the following morning the supernatant was aspirated. One spheroid (100–200 μm) was aspirated into a 0.1–2.0 μl pipette and placed on the coating. After incubating the plate for 75 minutes at 36° C, 1000 μl serum-free medium was added. The migration was followed with time-lapse microscopy for 5 days. Migration speed was calculated to find the optimal time to isolate the migrating cells. At the highest migration speed, the migrating cells were isolated by removing the “central” residual spheroid with a micro-pipette. The migrating cells were then washed twice in phosphate buffered saline (PBS). Migrating cell pellets were stored at −80°C until further proteomic analysis was performed. For comparison with non-migrating cells, pellets from free-floating spheroids were obtained.

### Sample preparation for quantitative proteomics

We used three independent replicates per GBM subpopulation in this study. Cells (about 200000 for each cell type) were lysed and proteins were on-filter digested as previously published [65]. Briefly, GBM cells were lysed with a solution of 2% (w/v) sodium dodecyl sulfate (SDS), 20 mmol/L triethylammonium bicarbonate (TEAB), 0.1 mol/L dithiothreitol (DTT), phosphatase inhibitor (PhosSTOP, Roche, Switzerland), and protease inhibitor (cOmplete, Roche, Switzerland). Lysis was enhanced and DNA filaments sheared with tip sonication on ice. Protein concentration was measured using Qubit assay (Thermo Fisher Scientific, USA) as μg/μL. Proteins were loaded onto spin-filter units (Vivacon 500, 30,000 MWCO; Vivaproducts, USA) and the SDS-containing solution was washed out using an urea-containing solution (8 mol/L urea, 20 mmol/L TEAB). The lysate was loaded in three steps, 300 μL of urea solution was used for washing after each step and two washes with 600 μL of 1% (w/v) sodium deoxycholate (SDC), 20 mmol/L TEAB after all loadings. Alkylation of the reduced thiol groups was done with 50 mmol/L iodoacetamide, 1% (w/v) SDC, 20 mmol/L TEAB, followed by two times wash with 250 μL of 1% (w/v) SDC, 20 mmol/L TEAB, proteins were digested overnight with trypsin (1:50) (Promega, USA) in 1% (w/v) SDC, 20 mmol/L TEAB. Peptides were collected after centrifugation, and SDC was removed using ethyl acetate extraction from acidic solution (TFA (0.5% (v/v)).

Dimethyl labeling was performed according to a published protocol [66]. Briefly, 22.8 μg of peptides per labeling channel were dissolved in 100 μL of 0.1 mol/L TEAB. Peptide amount was measured by the amino acid analyzer (Biocrom 30, Biochrom, UK). Next, 4 μL of 4% (vol/vol) solution of CH_2_O, CD_2_O, or ^13^CD_2_O was added and the samples were vortexed. Later, 4 μL of 0.6 mol/L NaBH_3_CN or NaBD_3_CN was added and the mixture was incubated for 60 min. at room temperature. The efficiency of labeling was monitored by liquid chromatography coupled to mass spectrometry (LC-MS) run before quenching the reaction. The reaction was quenched by adding 16 μL of 1% (vol/vol) ammonia solution and later 8 μL of 5% (vol/vol) formic acid. After quenching labelled peptides were mixed in 1:1:1 ratio resulting in 3 differently labeled samples (Table 3). The samples were dried in a SpeedVac and stored at −20° C until analyzed by LC-MS.

**Table 3:** Labeling scheme for GBM cells

	Light (+ 28 Da)	Medium (+32 Da)	Heavy (+36 Da)
**Replicate 1**	Differentiated	Migrating	Spheroid
**Replicate 2**	Migrating	Spheroid	Differentiated
**Replicate 3**	Spheroid	Differentiated	Migrating

### LC-MS

Peptides were separated using Dionex Ultimate 3000 nanoUPLC system coupled to Thermo Orbitrap Fusion mass spectrometer. Peptides were focused on the precolumn (PepMap C18 10 cm × 150 μm i.d., 5 μm) and eluted from analytical column (PepMap C18 50 cm × 75 μm i.d., 3 μm) with the gradient presented in Table 4. The mass spectrometer was configured to run in top speed mode with 3 seconds cycle duration. MS^1^ spectra were recorded in Orbitrap mass analyzer from 400 to 1200 Th, with 120000 resolution at 200 Th, AGC target value–5e5, maximum accumulation time–60 ms. Ions were isolated using quadrupole mass filter with 2 Th wide isolation window and fragmented using CID in the ion trap, spectra were acquired in Orbitrap, with 15,000 resolution at 200 Th, AGC target–1e4, maximum accumulation time–40 ms.

**Table 4:** LC gradient used for the analysis

**Time (min)**	0	5	25	205	245	270	285	287	300
**% B**	2	2	5	21	35	99	99	2	2

A: 0.1% formic acid; B: 80% acetonitrile in 0.1% formic acid.

### Data analysis

Data analysis was performed using Thermo Proteome Discoverer 2.0.0.644. Mascot 2.3 was used as the database search engine. SwissProt database (2014.04) restricted to *Homo sapiens* (20,340 protein sequences) combined with common contaminants database (231 protein sequences) was used. Search parameters were: parent ion mass tolerance–5 ppm, fragment ion mass tolerance–0.02 Th; fixed modifications–carboxamidomethylated cysteine, variable modifications–oxidized methionine and labeled N-terminal and lysine. Reversed decoy database was searched separately. For SuperQuant analysis, all MS^2^ spectra were processed using home-built deconvolution node to produce fragmentation spectra consisting only of 1+ fragments. Next, deconvoluted spectra were processed with ComplementaryFinder node before database search. A detailed explanation of SuperQuant data processing is published [14]. Database search results were evaluated using Percolator 2.05 [67], with the standard feature set. All PSMs with *q*-value < 0.01 were grouped together using sequence and theoretical mass, and the highest Percolator SVM score was used as the score for the group. Qvality 2.05 [68] was used for the estimation of *q*-value on the PSM group level, PSM groups were filtered by *q*-value < 0.01. Each PSM group gives rise to one peptide. Proteins related to the filtered peptides were grouped using maximum parsimony principle. Quantification of peptides and proteins was performed using standard settings provided by Proteome Discoverer. The mass spectrometry proteomics data were deposited to the ProteomeXchange Consortium (http://proteomecentral.proteomexchange.org) via the PRIDE [69] partner repository with the dataset identifier PXD005245.

Peptides abundancies reported by Proteome Discoverer were log2 transformed, normalized by subtracting the mean, and protein rollup was performed using RRollup method from DanteR package [70]. Log2-transformed intensities were used for hierarchical clustering and CV calculation prior to normalization. Significantly regulated proteins were detected using limma R package as described earlier [71, 72] .

The software Ingenuity Pathway Analysis (IPA, Qiagen) was used to retrieve biologic and canonical functions. We used default settings except for the following instances: species = human; tissue & cell lines = astrocytes, microglia, neurons (all), stem cells (all), nervous system (all), CNS cell lines (all), neuroblastoma cell lines (all), tissues, primary cells and cells not otherwise specified; experimental *p*-value cutoff = 0.05.

### Immunohistochemistry

Immunohistochemical staining was performed on tissue samples matching the patient from whom T78 was derived. Briefly, formaldehyde fixed and paraffin embedded tissue was cut into 3 μm serial cut sections and placed on glass slides. The first section was stained with haematoxylin-eosin to define tumor core and tumor periphery. Four sections were dewaxed, and heat-induced epitope retrieval followed by quenching of endogen peroxidase was performed. Sections were then incubated with primary antibodies against vinculin (VCL) (1 + 800), procollagen-lysine, 2-oxoglutarate 5-dioxygenase 2 (PLOD2) (1 + 200), pleiotrophin (PTN) (1 + 400), and transferrin (TF) (1 + 800). Detection and staining procedures were done using EnVision (Dako) and the AutostainerPlus (Dako), respectively.

### Image analysis

Stained sections were evaluated using the Visiopharm software (Visiopharm, Hørsholm, Denmark). The five stainings were aligned using the TISSUEalign module (Visiopharm). Sample images were then collected for 14 regions (7 areas of tumor core and 7 areas of tumor periphery) using systematic uniform random sampling (meander fraction based) at a sampling fraction that resulted in approximately 5 images per region. To ensure optimal alignment, sample images were reviewed, and the two best-aligned images for each region were included for further analysis. Using the newCAST module (Visiopharm), tumor cells were counted manually to estimate the number of positive and negative tumor cells in each staining separately. Positive cell fractions were calculated on the basis of the total tumor cell number.

## SUPPLEMENTARY MATERIALS FIGURES AND TABLES





## References

[1] Louis DN, Ohgaki H, Wiestler OD, Cavenee WK, Burger PC, Jouvet A, Scheithauer BW, Kleihues P (2007). The 2007 WHO classification of tumours of the central nervous system. Acta Neuropathol.

[2] Stupp R, Hegi ME, Mason WP, van den Bent MJ, Taphoorn MJ, Janzer RC, Ludwin SK, Allgeier A, Fisher B, Belanger K, Hau P, Brandes AA, Gijtenbeek J, and European Organisation for Research and Treatment of Cancer Brain Tumour and Radiation Oncology Groups, and National Cancer Institute of Canada Clinical Trials Group (2009). Effects of radiotherapy with concomitant and adjuvant temozolomide versus radiotherapy alone on survival in glioblastoma in a randomised phase III study: 5-year analysis of the EORTC-NCIC trial. Lancet Oncol.

[3] Bao S, Wu Q, McLendon RE, Hao Y, Shi Q, Hjelmeland AB, Dewhirst MW, Bigner DD, Rich JN (2006). Glioma stem cells promote radioresistance by preferential activation of the DNA damage response. Nature.

[4] Jun HJ, Bronson RT, Charest A (2014). Inhibition of EGFR induces a c-MET-driven stem cell population in glioblastoma. Stem Cells.

[5] Chen J, McKay RM, Parada LF (2012). Malignant glioma: lessons from genomics, mouse models, and stem cells. Cell.

[6] Lathia JD, Mack SC, Mulkearns-Hubert EE, Valentim CL, Rich JN (2015). Cancer stem cells in glioblastoma. Genes Dev.

[7] Noushmehr H, Weisenberger DJ, Diefes K, Phillips HS, Pujara K, Berman BP, Pan F, Pelloski CE, Sulman EP, Bhat KP, Verhaak RG, Hoadley KA, Ha yes DN, and Cancer Genome Atlas Research Network (2010). Identification of a CpG island methylator phenotype that defines a distinct subgroup of glioma. Cancer Cell.

[8] Yan H, Parsons DW, Jin G, McLendon R, Rasheed BA, Yuan W, Kos I, Batinic-Haberle I, Jones S, Riggins GJ, Friedman H, Friedman A, Reardon D (2009). IDH1 and IDH2 mutations in gliomas. N Engl J Med.

[9] Munthe S, Petterson SA, Dahlrot RH, Poulsen FR, Hansen S, Kristensen BW (2016). Glioma Cells in the Tumor Periphery Have a Stem Cell Phenotype. PLoS One.

[10] Hemmati HD, Nakano I, Lazareff JA, Masterman-Smith M, Geschwind DH, Bronner-Fraser M, Kornblum HI (2003). Cancerous stem cells can arise from pediatric brain tumors. Proc Natl Acad Sci U S A.

[11] Tunici P, Bissola L, Lualdi E, Pollo B, Cajola L, Broggi G, Sozzi G, Finocchiaro G (2004). Genetic alterations and *in vivo* tumorigenicity of neurospheres derived from an adult glioblastoma. Mol Cancer.

[12] Huang Z, Cheng L, Guryanova OA, Wu Q, Bao S (2010). Cancer stem cells in glioblastoma--molecular signaling and therapeutic targeting. Protein Cell.

[13] Kryuchkov F, Verano-Braga T, Hansen TA, Sprenger RR, Kjeldsen F (2013). Deconvolution of mixture spectra and increased throughput of peptide identification by utilization of intensified complementary ions formed in tandem mass spectrometry. J Proteome Res.

[14] Gorshkov V, Verano-Braga T, Kjeldsen F (2015). SuperQuant: A Data Processing Approach to Increase Quantitative Proteome Coverage. Anal Chem.

[15] Favicchio R, Thepaut C, Zhang H, Arends R, Stebbing J, Giamas G (2016). Strategies in functional proteomics: Unveiling the pathways to precision oncology. Cancer Lett.

[16] Jensen SS, Aaberg-Jessen C, Andersen C, Schroder HD, Kristensen BW (2013). Glioma spheroids obtained via ultrasonic aspiration are viable and express stem cell markers: a new tissue resource for glioma research. Neurosurgery.

[17] Bjerkvig R, Tonnesen A, Laerum OD, Backlund EO (1990). Multicellular tumor spheroids from human gliomas maintained in organ culture. J Neurosurg.

[18] Christensen K, Aaberg-Jessen C, Andersen C, Goplen D, Bjerkvig R, Kristensen BW (2010). Immunohistochemical expression of stem cell, endothelial cell, and chemosensitivity markers in primary glioma spheroids cultured in serum-containing and serum-free medium. Neurosurgery.

[19] Munthe S, Sorensen MD, Thomassen M, Burton M, Kruse TA, Lathia JD, Poulsen FR, Kristensen BW (2016). Migrating glioma cells express stem cell markers and give rise to new tumors upon xenografting. J Neurooncol.

[20] Gilbert CA, Ross AH (2009). Cancer stem cells: cell culture, markers, and targets for new therapies. J Cell Biochem.

[21] Lee J, Kotliarova S, Kotliarov Y, Li A, Su Q, Donin NM, Pastorino S, Purow BW, Christopher N, Zhang W, Park JK, Fine HA (2006). Tumor stem cells derived from glioblastomas cultured in bFGF and EGF more closely mirror the phenotype and genotype of primary tumors than do serum-cultured cell lines. Cancer Cell.

[22] Kramer A, Green J, Pollard J, Tugendreich S (2014). Causal analysis approaches in Ingenuity Pathway Analysis. Bioinformatics.

[23] McDonald PC, Fielding AB, Dedhar S (2008). Integrin-linked kinase--essential roles in physiology and cancer biology. J Cell Sci.

[24] Hausmann C, Temme A, Cordes N, Eke I (2015). ILKAP, ILK and PINCH1 control cell survival of p53-wildtype glioblastoma cells after irradiation. Oncotarget.

[25] Artym VV, Zhang Y, Seillier-Moiseiwitsch F, Yamada KM, Mueller SC (2006). Dynamic interactions of cortactin and membrane type 1 matrix metalloproteinase at invadopodia: defining the stages of invadopodia formation and function. Cancer Res.

[26] Mallawaaratchy DM, Buckland ME, McDonald KL, Li CC, Ly L, Sykes EK, Christopherson RI, Kaufman KL (2015). Membrane proteome analysis of glioblastoma cell invasion. J Neuropathol Exp Neurol.

[27] Bleau AM, Hambardzumyan D, Ozawa T, Fomchenko EI, Huse JT, Brennan CW, Holland EC (2009). PTEN/PI3K/Akt pathway regulates the side population phenotype and ABCG2 activity in glioma tumor stem-like cells. Cell Stem Cell.

[28] Meister G (2007). miRNAs get an early start on translational silencing. Cell.

[29] Fowler A, Thomson D, Giles K, Maleki S, Mreich E, Wheeler H, Leedman P, Biggs M, Cook R, Little N, Robinson B, McDonald K (2011). miR-124a is frequently down-regulated in glioblastoma and is involved in migration and invasion. Eur J Cancer.

[30] Ludwig N, Leidinger P, Becker K, Backes C, Fehlmann T, Pallasch C, Rheinheimer S, Meder B, Stahler C, Meese E, Keller A (2016). Distribution of miRNA expression across human tissues. Nucleic Acids Res.

[31] Kanaan Z, Rai SN, Eichenberger MR, Barnes C, Dworkin AM, Weller C, Cohen E, Roberts H, Keskey B, Petras RE, Crawford NP, Galandiuk S (2012). Differential microRNA expression tracks neoplastic progression in inflammatory bowel disease-associated colorectal cancer. Hum Mutat.

[32] Kunte DP, DelaCruz M, Wali RK, Menon A, Du H, Stypula Y, Patel A, Backman V, Roy HK (2012). Dysregulation of microRNAs in colonic field carcinogenesis: implications for screening. PLoS One.

[33] Wang B, Wang H, Yang Z (2012). MiR-122 inhibits cell proliferation and tumorigenesis of breast cancer by targeting IGF1R. PLoS One.

[34] Wang G, Zhao Y, Zheng Y (2014). MiR-122/Wnt/beta-catenin regulatory circuitry sustains glioma progression. Tumour Biol.

[35] Nakao K, Miyaaki H, Ichikawa T (2014). Antitumor function of microRNA-122 against hepatocellular carcinoma. J Gastroenterol.

[36] Meng Q, Xing Y, Ren T, Lu H, Xi Y, Jiang Z, Hu J, Li C, Sun L, Sun D, Cai L (2017). Mammalian Eps15 homology domain 1 promotes metastasis in non-small cell lung cancer by inducing epithelial-mesenchymal transition. Oncotarget.

[37] Dai B, Hu Z, Huang H, Zhu G, Xiao Z, Wan W, Zhang P, Jia W, Zhang L (2014). Overexpressed KDM5B is associated with the progression of glioma and promotes glioma cell growth via downregulating p21. Biochem Biophys Res Commun.

[38] Bamodu OA, Huang WC, Lee WH, Wu A, Wang LS, Hsiao M, Yeh CT, Chao TY (2016). Aberrant KDM5B expression promotes aggressive breast cancer through MALAT1 overexpression and downregulation of hsa-miR-448. BMC Cancer.

[39] Zhao C, Shen Y, Tao X, Xu J, Lu J, Liu C, Xu Z, Tang Q, Tao T, Zhang X (2016). Silencing of CtBP1 suppresses the migration in human glioma cells. J Mol Histol.

[40] Flavahan WA, Wu Q, Hitomi M, Rahim N, Kim Y, Sloan AE, Weil RJ, Nakano I, Sarkaria JN, Stringer BW, Day BW, Li M, Lathia JD (2013). Brain tumor initiating cells adapt to restricted nutrition through preferential glucose uptake. Nat Neurosci.

[41] Miyamoto K, Seki N, Matsushita R, Yonemori M, Yoshino H, Nakagawa M, Enokida H (2016). Tumour-suppressive miRNA-26a-5p and miR-26b-5p inhibit cell aggressiveness by regulating PLOD2 in bladder cancer. Br J Cancer.

[42] Kurozumi A, Kato M, Goto Y, Matsushita R, Nishikawa R, Okato A, Fukumoto I, Ichikawa T, Seki N (2016). Regulation of the collagen cross-linking enzymes LOXL2 and PLOD2 by tumor-suppressive microRNA-26a/b in renal cell carcinoma. Int J Oncol.

[43] Lu KV, Jong KA, Kim GY, Singh J, Dia EQ, Yoshimoto K, Wang MY, Cloughesy TF, Nelson SF, Mischel PS (2005). Differential induction of glioblastoma migration and growth by two forms of pleiotrophin. J Biol Chem.

[44] Muller S, Kunkel P, Lamszus K, Ulbricht U, Lorente GA, Nelson AM, von Schack D, Chin DJ, Lohr SC, Westphal M, Melcher T (2003). A role for receptor tyrosine phosphatase zeta in glioma cell migration. Oncogene.

[45] Cahoy JD, Emery B, Kaushal A, Foo LC, Zamanian JL, Christopherson KS, Xing Y, Lubischer JL, Krieg PA, Krupenko SA, Thompson WJ, Barres BA (2008). A transcriptome database for astrocytes, neurons, and oligodendrocytes: a new resource for understanding brain development and function. J Neurosci.

[46] Connor JR, Menzies SL, St Martin SM, Mufson EJ (1990). Cellular distribution of transferrin, ferritin, and iron in normal and aged human brains. J Neurosci Res.

[47] Schonberg DL, Miller TE, Wu Q, Flavahan WA, Das NK, Hale JS, Hubert CG, Mack SC, Jarrar AM, Karl RT, Rosager AM, Nixon AM, Tesar PJ (2015). Preferential Iron Trafficking Characterizes Glioblastoma Stem-like Cells. Cancer Cell.

[48] Eisinger-Mathason TS, Zhang M, Qiu Q, Skuli N, Nakazawa MS, Karakasheva T, Mucaj V, Shay JE, Stangenberg L, Sadri N, Puré E, Yoon SS, Kirsch DG, Simon MC (2013). Hypoxia-dependent modification of collagen networks promotes sarcoma metastasis. Cancer Discov.

[49] Chang HY, Sneddon JB, Alizadeh AA, Sood R, West RB, Montgomery K, Chi JT, van de Rijn M, Botstein D, Brown PO (2004). Gene expression signature of fibroblast serum response predicts human cancer progression: similarities between tumors and wounds. PLoS Biol.

[50] Gilkes DM, Semenza GL, Wirtz D (2014). Hypoxia and the extracellular matrix: drivers of tumour metastasis. Nat Rev Cancer.

[51] Ulrich TA, de Juan Pardo EM, Kumar S (2009). The mechanical rigidity of the extracellular matrix regulates the structure, motility, and proliferation of glioma cells. Cancer Res.

[52] Noda T, Yamamoto H, Takemasa I, Yamada D, Uemura M, Wada H, Kobayashi S, Marubashi S, Eguchi H, Tanemura M, Umeshita K, Doki Y, Mori M (2012). PLOD2 induced under hypoxia is a novel prognostic factor for hepatocellular carcinoma after curative resection. Liver Int.

[53] Dong S, Nutt CL, Betensky RA, Stemmer-Rachamimov AO, Denko NC, Ligon KL, Rowitch DH, Louis DN (2005). Histology-based expression profiling yields novel prognostic markers in human glioblastoma. J Neuropathol Exp Neurol.

[54] Lively S, Schlichter LC (2013). The microglial activation state regulates migration and roles of matrix-dissolving enzymes for invasion. J Neuroinflammation.

[55] Ziegler WH, Liddington RC, Critchley DR (2006). The structure and regulation of vinculin. Trends Cell Biol.

[56] Li T, Guo H, Song Y, Zhao X, Shi Y, Lu Y, Hu S, Nie Y, Fan D, Wu K (2014). Loss of vinculin and membrane-bound beta-catenin promotes metastasis and predicts poor prognosis in colorectal cancer. Mol Cancer.

[57] Somiari RI, Sullivan A, Russell S, Somiari S, Hu H, Jordan R, George A, Katenhusen R, Buchowiecka A, Arciero C, Brzeski H, Hooke J, Shriver C (2003). High-throughput proteomic analysis of human infiltrating ductal carcinoma of the breast. Proteomics.

[58] Lifschitz-Mercer B, Czernobilsky B, Feldberg E, Geiger B (1997). Expression of the adherens junction protein vinculin in human basal and squamous cell tumors: relationship to invasiveness and metastatic potential. Hum Pathol.

[59] Meyer T, Brinck U (1997). Immunohistochemical detection of vinculin in human rhabdomyosarcomas. Gen Diagn Pathol.

[60] Wang Y, Kuramitsu Y, Ueno T, Suzuki N, Yoshino S, Iizuka N, Zhang X, Akada J, Oka M, Nakamura K (2012). Proteomic differential display identifies upregulated vinculin as a possible biomarker of pancreatic cancer. Oncol Rep.

[61] Li YS, Milner PG, Chauhan AK, Watson MA, Hoffman RM, Kodner CM, Milbrandt J, Deuel TF (1990). Cloning and expression of a developmentally regulated protein that induces mitogenic and neurite outgrowth activity. Science.

[62] Kadomatsu K, Muramatsu T (2004). Midkine and pleiotrophin in neural development and cancer. Cancer Lett.

[63] Verhaak RG, Hoadley KA, Purdom E, Wang V, Qi Y, Wilkerson MD, Miller CR, Ding L, Golub T, Mesirov JP, Alexe G, Lawrence M, O’Kelly M, and Cancer Genome Atlas Research Network (2010). Integrated genomic analysis identifies clinically relevant subtypes of glioblastoma characterized by abnormalities in PDGFRA, IDH1, EGFR, and NF1. Cancer Cell.

[64] Kolenda J, Jensen SS, Aaberg-Jessen C, Christensen K, Andersen C, Brunner N, Kristensen BW (2011). Effects of hypoxia on expression of a panel of stem cell and chemoresistance markers in glioblastoma-derived spheroids. J Neurooncol.

[65] Leon IR, Schwammle V, Jensen ON, Sprenger RR (2013). Quantitative assessment of in-solution digestion efficiency identifies optimal protocols for unbiased protein analysis. Mol Cell Proteomics.

[66] Boersema PJ, Raijmakers R, Lemeer S, Mohammed S, Heck AJ (2009). Multiplex peptide stable isotope dimethyl labeling for quantitative proteomics. Nat Protoc.

[67] Kall L, Canterbury JD, Weston J, Noble WS, MacCoss MJ (2007). Semi-supervised learning for peptide identification from shotgun proteomics datasets. Nat Methods.

[68] Kall L, Storey JD, Noble WS (2009). QVALITY: non-parametric estimation of q-values and posterior error probabilities. Bioinformatics.

[69] Vizcaino JA, Cote RG, Csordas A, Dianes JA, Fabregat A, Foster JM, Griss J, Alpi E, Birim M, Contell J, O’Kelly G, Schoenegger A, Ovelleiro D (2013). The PRoteomics IDEntifications (PRIDE) database and associated tools: status in 2013. Nucleic Acids Res.

[70] Taverner T, Karpievitch YV, Polpitiya AD, Brown JN, Dabney AR, Anderson GA, Smith RD (2012). DanteR: an extensible R-based tool for quantitative analysis of -omics data. Bioinformatics.

[71] Smyth GK (2004). Linear models and empirical bayes methods for assessing differential expression in microarray experiments. Stat Appl Genet Mol Biol.

[72] Schwammle V, Leon IR, Jensen ON (2013). Assessment and improvement of statistical tools for comparative proteomics analysis of sparse data sets with few experimental replicates. J Proteome Res.

