# Supplementary material for: SuperQuant-assisted comparative proteome analysis of glioblastoma subpopulations allows for identification of potential novel therapeutic targets and cell markers
Source: Oncotarget. 2018 Jan 25;9(10):9400–14. doi: 10.18632/oncotarget.24321 (PMC5823648; doi:10.18632/oncotarget.24321)
Supplement: Supplementary file 1 [file oncotarget-09-9400-s001.pdf]

SuperQuant-assisted comparative proteome analysis of glioblastoma subpopulations allows for identification of potential novel therapeutic targets and cell markers

SUPPLEMENTARY MATERIALS

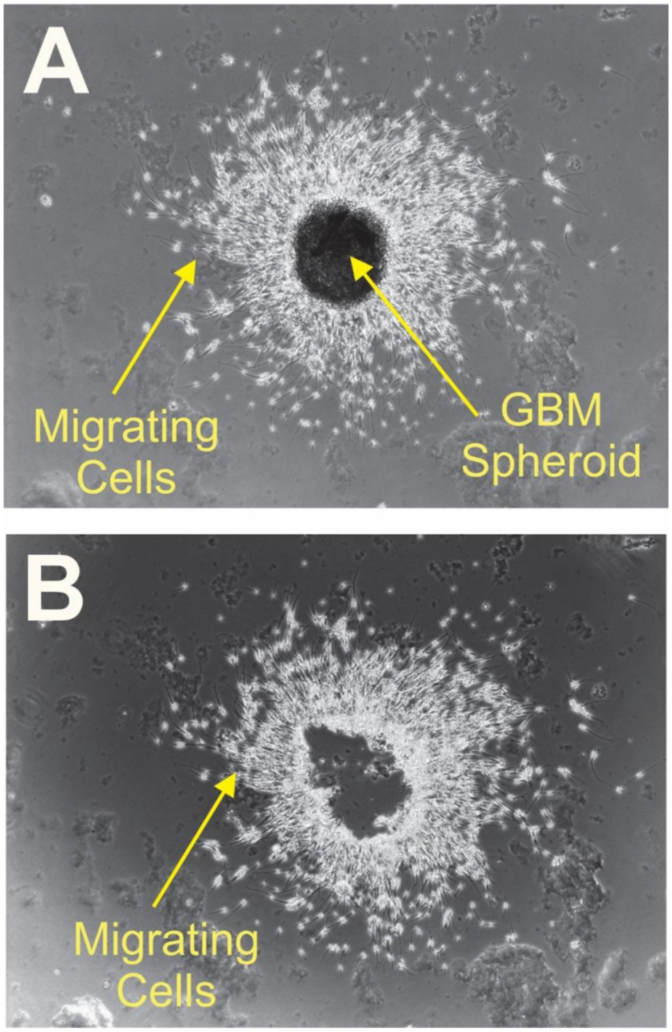

Supplementary Figure 1: Migration assay. (**A**) Spheroids were placed in coated wells using Geltrex and serum-free medium to preserve the stem-like phenotype. Migrating CSCs are observed in the periphery. (**B**) Core cells were removed to isolate migrating CSCs.

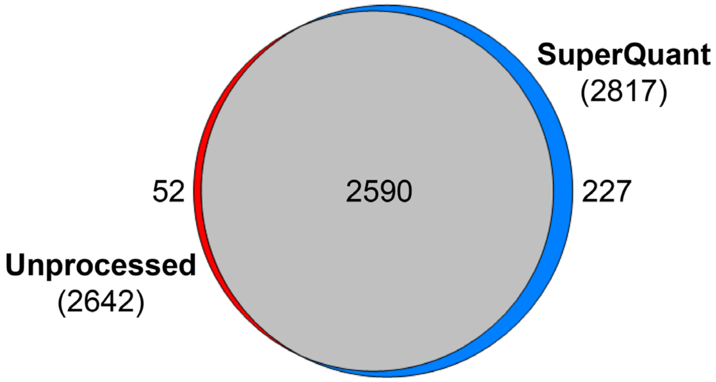

Supplementary Figure 2: Comparison of quantified proteins from unprocessed and SuperQuant processing. MS/MS spectra obtained from spheroid, migrating and differentiated cells were processed with SuperQuant or not.

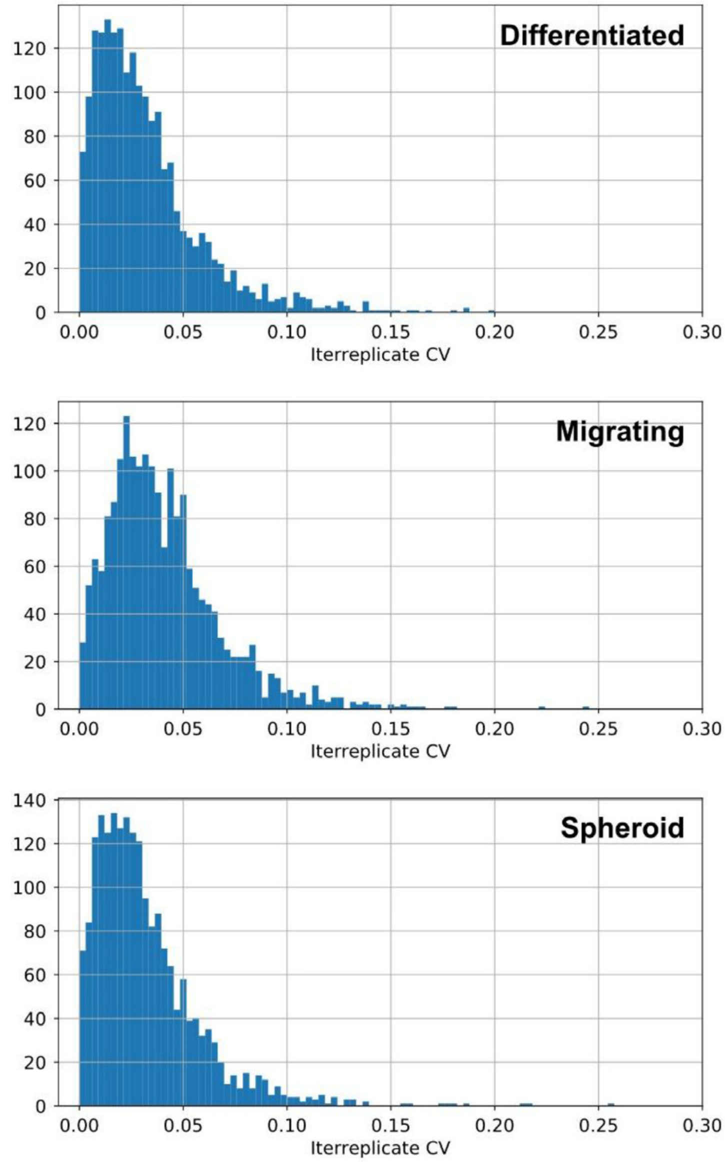

Supplementary Figure 3: Protein quantification reproducibility. To assess method reproducibility, CV between the 3 independent replicates was calculated for each quantified protein.

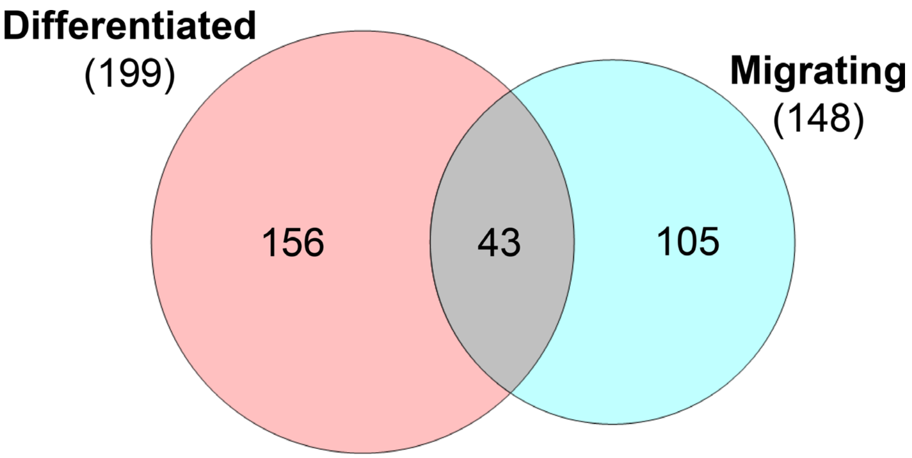

Supplementary Figure 4: Comparison of regulated datasets. Overlap of regulated proteins from migrating CSCs and differentiated cells.

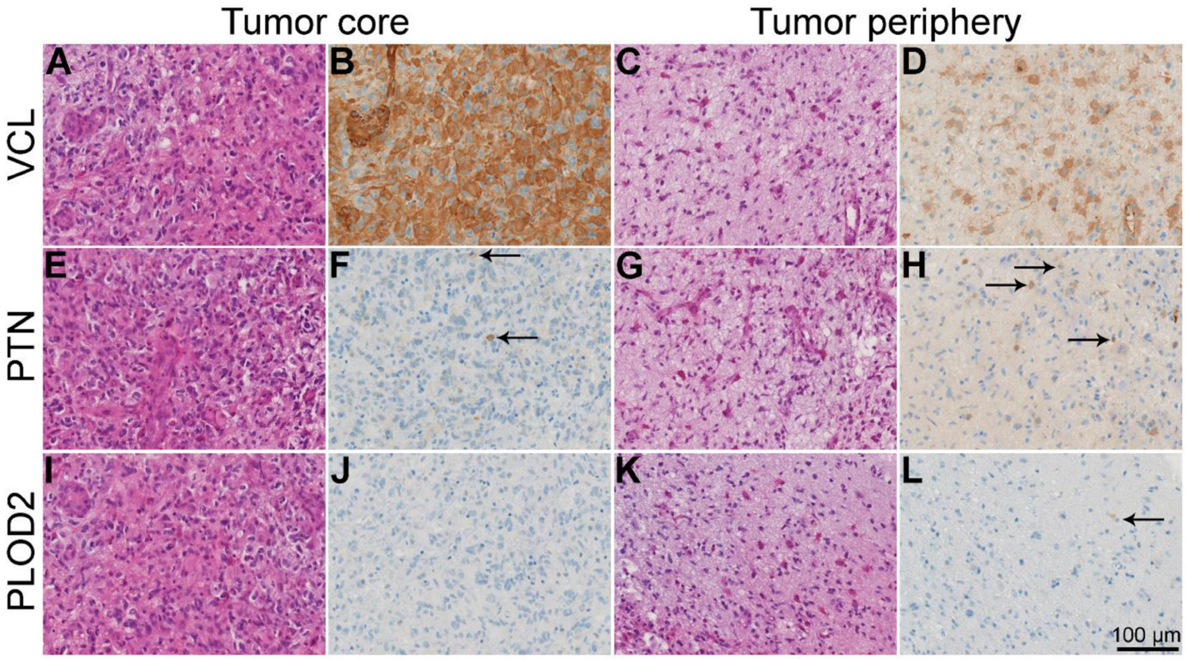

Supplementary Figure 5: Validation of VCL, PTN and PLOD2 using imunohistochemical staining of histological section. Tissues were stained with haematoxylin-eosin (HE) to define tumor core (**A**, **E** and **I**) and tumor periphery (**C**, **G** and **K**). Vinculin (VCL) expression was monitored in tumor core (**B**) and periphery (**D**) using anti-VCL. Pleiotrophin (PTN) expression was monitored in tumor core (**F**) and periphery (**H**) using anti-PTN. Procollagen-lysine, 2-oxoglutarate 5-dioxygenase 2 (PLOD2) expression was monitored in tumor core (**J**) and periphery (**L**) using anti-PLOD2.

Supplementary Table 1: List of regulated proteins from differentiated and migrating datasets. Proteins are sorted by their gene name. See Supplementary_Table_1
