# Supplementary material for: SuperQuant-assisted comparative proteome analysis of glioblastoma subpopulations allows for identification of potential novel therapeutic targets and cell markers
Source: Oncotarget. 2018 Jan 25;9(10):9400–14. doi: 10.18632/oncotarget.24321 (PMC5823648; doi:10.18632/oncotarget.24321)
Supplement: Supplementary file 2 [file oncotarget-09-9400-s002.docx]

**Supplementary Table 1: List of regulated proteins from differentiated and migrating datasets. Proteins are sorted by their gene name**

| Gene | ID | Diff/Spher | Migr/Spher | Diff/Spher (qval) | Migr/Spher (qval) | Location | Type(s) |
| --- | --- | --- | --- | --- | --- | --- | --- |
| ABAT | P80404 | -0.379 | -1.090 | 0.224 | 0.039 | Cytoplasm | enzyme |
| ADAM10 | O14672 | 1.257 | 1.970 | 0.113 | 0.041 | Plasma Membrane | peptidase |
| AGRN | O00468 | 0.069 | 1.501 | 0.499 | 0.049 | Plasma Membrane | other |
| AHCY | P23526 | -0.716 | 1.653 | 0.116 | 0.023 | Cytoplasm | enzyme |
| AHCYL2 | Q96HN2 | -1.871 | 0.455 | 0.013 | 0.259 | Other | enzyme |
| AHNAK | Q09666 | 1.489 | -0.401 | 0.034 | 0.302 | Nucleus | other |
| AIP | O00170 | -2.997 | -0.232 | 0.015 | 0.418 | Nucleus | transcription regulator |
| ALCAM | Q13740 | 2.366 | 0.778 | 0.024 | 0.235 | Plasma Membrane | other |
| ALDH7A1 | P49419 | -0.522 | -1.167 | 0.153 | 0.035 | Cytoplasm | enzyme |
| ALDOA | P04075 | -1.810 | -1.206 | 0.008 | 0.035 | Cytoplasm | enzyme |
| ALDOC | P09972 | -2.409 | -0.355 | 0.042 | 0.393 | Cytoplasm | enzyme |
| ANAPC1 | Q9H1A4 | -1.099 | 2.651 | 0.146 | 0.039 | Nucleus | other |
| ANKRD17 | O75179 | -4.066 | -1.347 | 0.044 | 0.206 | Nucleus | other |
| ANP32B | Q92688 | -2.626 | -1.854 | 0.015 | 0.047 | Nucleus | other |
| ANXA1 | P04083 | 1.787 | 0.836 | 0.029 | 0.167 | Plasma Membrane | enzyme |
| ANXA2 | P07355 | 3.382 | 0.995 | 0.006 | 0.162 | Plasma Membrane | other |
| ANXA4 | P09525 | 2.368 | 2.015 | 0.013 | 0.025 | Plasma Membrane | other |
| ANXA5 | P08758 | 1.808 | 0.969 | 0.013 | 0.071 | Plasma Membrane | transporter |
| AP2A1 | O95782 | -0.183 | 1.270 | 0.392 | 0.035 | Cytoplasm | transporter |
| AP3B1 | O00203 | 0.166 | 1.528 | 0.442 | 0.044 | Plasma Membrane | transporter |
| ARHGAP1 | Q07960 | -0.391 | 2.445 | 0.386 | 0.023 | Cytoplasm | other |
| ATIC | P31939 | -1.398 | -1.239 | 0.035 | 0.055 | Cytoplasm | enzyme |
| ATP1B1 | P05026 | 1.578 | 1.530 | 0.044 | 0.053 | Plasma Membrane | transporter |
| ATP2A2 | P16615 | 0.424 | 2.617 | 0.299 | 0.012 | Cytoplasm | transporter |
| ATP5E | P56381 | 0.286 | 1.163 | 0.337 | 0.041 | Cytoplasm | other |
| ATP6V0A1 | Q93050 | 1.718 | -0.065 | 0.044 | 0.485 | Cytoplasm | transporter |
| ATP6V0D1 | P61421 | 1.217 | 0.077 | 0.044 | 0.470 | Cytoplasm | transporter |
| BAG6 | P46379 | -0.028 | 1.898 | 0.522 | 0.039 | Nucleus | enzyme |
| BAIAP2 | Q9UQB8 | -3.978 | 0.523 | 0.044 | 0.375 | Plasma Membrane | kinase |
| BCAT1 | P54687 | -2.272 | -2.275 | 0.052 | 0.028 | Cytoplasm | enzyme |
| BET1 | O15155 | 2.454 | 0.731 | 0.042 | 0.296 | Cytoplasm | transporter |
| BRK1 | Q8WUW1 | -1.695 | -4.754 | 0.139 | 0.039 | Cytoplasm | other |
| BZW1 | Q7L1Q6 | -2.112 | -0.522 | 0.015 | 0.266 | Cytoplasm | translation regulator |
| C7orf50 | Q9BRJ6 | -5.499 | -1.887 | 0.036 | 0.220 | Other | other |
| CALM3 | P62158 | -1.143 | -1.787 | 0.055 | 0.023 | Cytoplasm | other |
| CAP1 | Q01518 | -1.771 | -1.153 | 0.022 | 0.073 | Plasma Membrane | other |
| CAV1 | Q03135 | 5.244 | 5.835 | 0.004 | 0.003 | Plasma Membrane | transmembrane receptor |
| CBX3 | Q13185 | -1.923 | -0.226 | 0.013 | 0.388 | Nucleus | transcription regulator |
| CCDC124 | Q96CT7 | -2.299 | -0.980 | 0.016 | 0.115 | Other | other |
| CCT8 | P50990 | -1.076 | -0.285 | 0.047 | 0.328 | Cytoplasm | enzyme |
| CD151 | P48509 | 0.835 | -3.321 | 0.343 | 0.047 | Plasma Membrane | other |
| CD44 | P16070 | 3.100 | 0.269 | 0.006 | 0.390 | Plasma Membrane | enzyme |
| CDC23 | Q9UJX2 | 0.489 | 4.117 | 0.380 | 0.047 | Nucleus | enzyme |
| CDK5RAP3 | Q96JB5 | 0.026 | -2.214 | 0.521 | 0.035 | Nucleus | other |
| CDS2 | O95674 | -0.578 | -1.319 | 0.154 | 0.035 | Cytoplasm | enzyme |
| CEND1 | Q8N111 | 1.458 | -0.266 | 0.028 | 0.358 | Other | other |
| CERS2 | Q96G23 | 1.642 | 2.629 | 0.090 | 0.041 | Nucleus | transcription regulator |
| CNDP2 | Q96KP4 | -1.440 | -0.250 | 0.047 | 0.393 | Cytoplasm | peptidase |
| COL4A1 | P02462 | -2.398 | 3.727 | 0.047 | 0.023 | Extracellular Space | other |
| COL4A2 | P08572 | -0.166 | 5.542 | 0.505 | 0.023 | Extracellular Space | other |
| COL6A1 | P12109 | 0.825 | 4.647 | 0.335 | 0.025 | Extracellular Space | other |
| COPB2 | P35606 | 0.220 | 1.713 | 0.407 | 0.035 | Cytoplasm | transporter |
| COPG1 | Q9Y678 | -0.079 | 1.537 | 0.484 | 0.031 | Cytoplasm | transporter |
| CORO1C | Q9ULV4 | -1.664 | -0.041 | 0.016 | 0.487 | Cytoplasm | other |
| CORO2B | Q9UQ03 | -1.628 | -0.916 | 0.045 | 0.162 | Other | other |
| CPNE1 | Q99829 | 0.784 | 1.205 | 0.077 | 0.035 | Nucleus | transporter |
| CPS1 | P31327 | 0.159 | 2.173 | 0.455 | 0.025 | Cytoplasm | enzyme |
| CPSF1 | Q10570 | 1.502 | 2.481 | 0.086 | 0.025 | Nucleus | other |
| CRYAB | P02511 | 5.230 | 0.768 | 0.002 | 0.241 | Nucleus | other |
| CSTF3 | Q12996 | -2.239 | -0.263 | 0.029 | 0.403 | Nucleus | other |
| CTBP1 | Q13363 | -0.429 | 2.800 | 0.375 | 0.025 | Nucleus | enzyme |
| CTNNA2 | P26232 | 1.397 | -0.380 | 0.046 | 0.317 | Plasma Membrane | other |
| DAZAP1 | Q96EP5 | -1.572 | -0.138 | 0.042 | 0.442 | Other | other |
| DBNL | Q9UJU6 | -1.426 | -2.658 | 0.059 | 0.023 | Cytoplasm | other |
| DCTN2 | Q13561 | -1.349 | -1.477 | 0.031 | 0.031 | Cytoplasm | other |
| DCXR | Q7Z4W1 | -1.141 | 0.719 | 0.028 | 0.091 | Cytoplasm | enzyme |
| DDX39B | Q13838 | -0.605 | -1.605 | 0.223 | 0.045 | Nucleus | enzyme |
| DDX42 | Q86XP3 | -2.281 | -0.516 | 0.013 | 0.270 | Cytoplasm | enzyme |
| DDX5 | P17844 | -2.120 | 0.623 | 0.013 | 0.213 | Nucleus | enzyme |
| DNAJB6 | O75190 | 1.632 | 4.212 | 0.199 | 0.047 | Nucleus | transcription regulator |
| DNAJC10 | Q8IXB1 | 0.277 | 1.190 | 0.330 | 0.041 | Cytoplasm | enzyme |
| DPYSL3 | Q14195 | -2.381 | -0.715 | 0.007 | 0.168 | Cytoplasm | enzyme |
| EEF1A1 | P68104 | -1.699 | -0.955 | 0.035 | 0.135 | Cytoplasm | translation regulator |
| EEF1B2 | P24534 | -1.625 | -0.822 | 0.029 | 0.168 | Cytoplasm | translation regulator |
| EEF1G | P26641 | -1.218 | -0.656 | 0.038 | 0.153 | Cytoplasm | translation regulator |
| EHD1 | Q9H4M9 | -0.292 | 1.975 | 0.408 | 0.047 | Cytoplasm | other |
| EIF2AK2 | P19525 | -0.609 | -1.488 | 0.206 | 0.047 | Cytoplasm | kinase |
| EIF2S3 | P41091 | -0.092 | 1.337 | 0.475 | 0.039 | Cytoplasm | translation regulator |
| EIF3A | Q14152 | -1.336 | 0.997 | 0.043 | 0.088 | Cytoplasm | other |
| EIF3CL | B5ME19 | -0.344 | 1.409 | 0.303 | 0.037 | Other | other |
| EIF3F | O00303 | -1.319 | 1.567 | 0.029 | 0.023 | Cytoplasm | translation regulator |
| EIF3H | O15372 | -0.337 | 2.781 | 0.375 | 0.020 | Cytoplasm | other |
| EIF3K | Q9UBQ5 | -1.649 | 0.532 | 0.028 | 0.247 | Cytoplasm | translation regulator |
| EIF3L | Q9Y262 | 0.083 | 2.181 | 0.482 | 0.012 | Cytoplasm | other |
| EIF4B | P23588 | -2.043 | -0.677 | 0.022 | 0.242 | Cytoplasm | translation regulator |
| EIF4G2 | P78344 | -1.474 | -0.754 | 0.033 | 0.155 | Cytoplasm | translation regulator |
| ELAVL1 | Q15717 | -1.739 | -1.873 | 0.046 | 0.042 | Cytoplasm | other |
| EMC1 | Q8N766 | 1.043 | 1.818 | 0.080 | 0.025 | Plasma Membrane | other |
| EMC3 | Q9P0I2 | 1.304 | 0.578 | 0.046 | 0.220 | Nucleus | other |
| ETF1 | P62495 | -1.494 | -0.488 | 0.038 | 0.270 | Cytoplasm | translation regulator |
| EZR | P15311 | 0.311 | -1.044 | 0.279 | 0.043 | Plasma Membrane | other |
| FABP5 | Q01469 | -2.143 | -2.205 | 0.038 | 0.034 | Cytoplasm | transporter |
| FABP7 | O15540 | -1.846 | -0.473 | 0.048 | 0.312 | Cytoplasm | transporter |
| FAM186B | Q8IYM0 | 1.236 | -0.045 | 0.034 | 0.485 | Other | other |
| FARSB | Q9NSD9 | -1.912 | -0.476 | 0.022 | 0.253 | Cytoplasm | enzyme |
| FBL | P22087 | -0.177 | 1.250 | 0.411 | 0.045 | Nucleus | other |
| FERMT2 | Q96AC1 | -1.381 | 2.429 | 0.068 | 0.025 | Cytoplasm | other |
| FKBP3 | Q00688 | -3.370 | -2.325 | 0.013 | 0.041 | Nucleus | enzyme |
| FMR1 | Q06787 | -1.607 | -0.420 | 0.024 | 0.287 | Nucleus | other |
| FN1 | P02751 | 0.486 | 2.924 | 0.371 | 0.044 | Extracellular Space | enzyme |
| FNTA | P49354 | -2.371 | -1.665 | 0.017 | 0.046 | Cytoplasm | enzyme |
| FSCN1 | Q16658 | -1.366 | -1.045 | 0.047 | 0.091 | Cytoplasm | other |
| FUBP1 | Q96AE4 | -1.998 | -2.131 | 0.013 | 0.014 | Nucleus | transcription regulator |
| FXR2 | P51116 | -2.223 | -4.565 | 0.101 | 0.041 | Cytoplasm | other |
| G3BP1 | Q13283 | -0.623 | -1.427 | 0.151 | 0.035 | Nucleus | enzyme |
| GBF1 | Q92538 | 0.559 | 2.377 | 0.335 | 0.041 | Cytoplasm | other |
| GDI1 | P31150 | -2.334 | -1.982 | 0.036 | 0.060 | Cytoplasm | other |
| GDI2 | P50395 | -1.878 | -1.138 | 0.022 | 0.080 | Cytoplasm | other |
| GFAP | P14136 | 2.699 | -0.170 | 0.012 | 0.441 | Cytoplasm | other |
| GLUL | P15104 | -4.353 | -3.020 | 0.010 | 0.039 | Cytoplasm | enzyme |
| GLYR1 | Q49A26 | -0.631 | 2.999 | 0.327 | 0.035 | Other | other |
| GM2A | P17900 | -0.955 | -1.729 | 0.092 | 0.039 | Cytoplasm | enzyme |
| GNB2L1 | P63244 | -0.720 | 1.535 | 0.158 | 0.041 | Cytoplasm | enzyme |
| GNPDA1 | P46926 | -2.073 | -0.839 | 0.040 | 0.202 | Cytoplasm | enzyme |
| GOLM1 | Q8NBJ4 | 1.537 | 0.463 | 0.033 | 0.287 | Cytoplasm | other |
| GPC4 | O75487 | 1.541 | -0.177 | 0.044 | 0.434 | Plasma Membrane | transmembrane receptor |
| GPI | P06744 | -2.487 | -2.439 | 0.004 | 0.007 | Extracellular Space | enzyme |
| GPX8 | Q8TED1 | 2.734 | 0.681 | 0.044 | 0.306 | Cytoplasm | enzyme |
| GSTO1 | P78417 | -2.171 | -3.255 | 0.043 | 0.023 | Cytoplasm | enzyme |
| GSTP1 | P09211 | -1.433 | -1.044 | 0.014 | 0.041 | Cytoplasm | enzyme |
| GTPBP4 | Q9BZE4 | 0.555 | 1.766 | 0.250 | 0.041 | Nucleus | enzyme |
| GYS1 | P13807 | -1.631 | 1.069 | 0.044 | 0.119 | Cytoplasm | enzyme |
| HBA2 | P69905 | 5.718 | 3.878 | 0.022 | 0.057 | Extracellular Space | transporter |
| HIBCH | Q6NVY1 | -2.652 | -2.948 | 0.044 | 0.059 | Cytoplasm | enzyme |
| HIST1H1E | P10412 | -2.135 | 0.354 | 0.023 | 0.341 | Nucleus | other |
| HIST1H2BA | Q96A08 | -1.019 | 0.550 | 0.047 | 0.173 | Nucleus | other |
| HIST1H3J | P68431 | -3.744 | -3.036 | 0.049 | 0.074 | Nucleus | other |
| HMGB1 | P09429 | -1.682 | -1.501 | 0.023 | 0.039 | Nucleus | transcription regulator |
| HMGB2 | P26583 | -1.843 | -0.782 | 0.031 | 0.192 | Nucleus | transcription regulator |
| HNRNPF | P52597 | -1.599 | -0.635 | 0.030 | 0.206 | Nucleus | other |
| HNRNPR | O43390 | -1.307 | -0.485 | 0.016 | 0.175 | Nucleus | other |
| HSPA4 | P34932 | -1.478 | -0.375 | 0.033 | 0.312 | Cytoplasm | other |
| IARS2 | Q9NSE4 | -0.327 | -1.294 | 0.294 | 0.036 | Cytoplasm | enzyme |
| IDH1 | O75874 | -1.829 | -1.145 | 0.013 | 0.055 | Cytoplasm | enzyme |
| IKBIP | Q70UQ0 | 1.901 | 0.235 | 0.022 | 0.403 | Cytoplasm | other |
| INS-IGF2 | F8WCM5 | -2.599 | 2.919 | 0.026 | 0.025 | Other | other |
| IPO9 | Q96P70 | -1.252 | 0.676 | 0.042 | 0.159 | Nucleus | transporter |
| ITGB8 | P26012 | 1.583 | -0.467 | 0.040 | 0.295 | Plasma Membrane | other |
| KHDRBS1 | Q07666 | -1.459 | -0.627 | 0.023 | 0.168 | Nucleus | transcription regulator |
| KHSRP | Q92945 | -1.772 | -0.564 | 0.030 | 0.260 | Nucleus | enzyme |
| KRT2 | P35908 | 1.814 | 2.316 | 0.013 | 0.007 | Cytoplasm | other |
| LAMA1 | P25391 | -1.120 | 4.505 | 0.307 | 0.035 | Extracellular Space | other |
| LAMB1 | P07942 | -0.042 | 4.188 | 0.521 | 0.013 | Extracellular Space | other |
| LAMC1 | P11047 | -1.666 | 1.963 | 0.029 | 0.023 | Extracellular Space | other |
| LAMP2 | P13473 | 1.248 | -2.262 | 0.047 | 0.012 | Plasma Membrane | enzyme |
| LARP4B | Q92615 | -5.728 | -3.337 | 0.026 | 0.064 | Cytoplasm | other |
| LASP1 | Q14847 | -1.128 | -0.643 | 0.033 | 0.151 | Cytoplasm | transporter |
| LDHA | P00338 | -1.611 | -1.051 | 0.013 | 0.047 | Cytoplasm | enzyme |
| LDHB | P07195 | -2.062 | 1.505 | 0.013 | 0.037 | Cytoplasm | enzyme |
| LGALS3 | P17931 | -1.509 | -0.321 | 0.015 | 0.297 | Extracellular Space | other |
| LIPA | P38571 | -0.784 | -2.090 | 0.220 | 0.043 | Cytoplasm | enzyme |
| LONP1 | P36776 | 0.087 | -1.118 | 0.463 | 0.035 | Cytoplasm | peptidase |
| LRRFIP1 | Q32MZ4 | -1.797 | -0.533 | 0.016 | 0.287 | Cytoplasm | other |
| MANF | P55145 | -0.167 | -1.257 | 0.410 | 0.039 | Extracellular Space | other |
| MAP1LC3B2 | A6NCE7 | -0.540 | -4.600 | 0.368 | 0.041 | Other | other |
| MAP2 | P11137 | -1.670 | -0.095 | 0.013 | 0.451 | Plasma Membrane | other |
| MAPK1 | P28482 | -1.784 | -0.470 | 0.022 | 0.278 | Cytoplasm | kinase |
| MARCKSL1 | P49006 | -1.724 | -1.716 | 0.015 | 0.023 | Cytoplasm | other |
| MCAM | P43121 | 1.739 | 0.140 | 0.044 | 0.452 | Plasma Membrane | other |
| MCM6 | Q14566 | -1.346 | 0.176 | 0.044 | 0.421 | Nucleus | enzyme |
| MOB4 | Q9Y3A3 | -1.277 | 1.690 | 0.076 | 0.049 | Cytoplasm | other |
| MRPL38 | Q96DV4 | 1.425 | 1.680 | 0.050 | 0.039 | Cytoplasm | other |
| MRPL53 | Q96EL3 | 1.796 | 1.397 | 0.016 | 0.077 | Cytoplasm | other |
| MRPS26 | Q9BYN8 | 0.673 | -1.770 | 0.176 | 0.038 | Cytoplasm | other |
| MTA2 | O94776 | 0.390 | 2.221 | 0.284 | 0.012 | Nucleus | transcription regulator |
| MTPN | P58546 | -2.515 | -2.505 | 0.022 | 0.030 | Nucleus | transcription regulator |
| MTX1 | Q13505 | 1.582 | -0.075 | 0.027 | 0.476 | Cytoplasm | transporter |
| MYH14 | Q7Z406 | 3.409 | 2.259 | 0.013 | 0.041 | Extracellular Space | other |
| MYL1 | P05976 | 1.809 | 2.221 | 0.050 | 0.038 | Cytoplasm | other |
| MYL6 | P60660 | 1.343 | -0.044 | 0.029 | 0.485 | Cytoplasm | other |
| MYO1C | O00159 | 1.662 | 1.066 | 0.036 | 0.121 | Cytoplasm | other |
| MYOF | Q9NZM1 | 2.704 | -0.515 | 0.015 | 0.319 | Nucleus | other |
| NACA | E9PAV3 | -2.197 | -0.430 | 0.018 | 0.324 | Cytoplasm | transcription regulator |
| NAP1L1 | P55209 | -1.632 | -4.582 | 0.146 | 0.041 | Nucleus | other |
| NCALD | P61601 | -4.127 | -2.771 | 0.043 | 0.080 | Cytoplasm | other |
| NCEH1 | Q6PIU2 | 2.682 | -0.579 | 0.002 | 0.134 | Plasma Membrane | enzyme |
| NDUFAF7 | Q7L592 | -0.717 | -1.593 | 0.162 | 0.045 | Cytoplasm | other |
| NDUFS8 | O00217 | 0.465 | 2.217 | 0.257 | 0.023 | Cytoplasm | enzyme |
| NME1 | P15531 | -2.570 | -1.523 | 0.039 | 0.134 | Cytoplasm | kinase |
| NME2 | P22392 | -1.525 | -2.330 | 0.044 | 0.020 | Nucleus | kinase |
| NOC3L | Q8WTT2 | 0.400 | 2.898 | 0.375 | 0.025 | Nucleus | other |
| NOVA1 | P51513 | -1.571 | -1.299 | 0.045 | 0.076 | Nucleus | other |
| NOVA2 | Q9UNW9 | -0.481 | -1.783 | 0.260 | 0.039 | Nucleus | other |
| NPC2 | P61916 | 0.093 | -2.774 | 0.483 | 0.007 | Extracellular Space | other |
| NPEPPS | P55786 | -1.834 | 0.137 | 0.013 | 0.432 | Cytoplasm | peptidase |
| NPTN | Q9Y639 | 0.703 | 1.606 | 0.183 | 0.042 | Plasma Membrane | other |
| NUCB2 | P80303 | -0.893 | -2.864 | 0.174 | 0.025 | Nucleus | other |
| NUDT21 | O43809 | -2.083 | -0.463 | 0.006 | 0.229 | Nucleus | other |
| OLA1 | Q9NTK5 | -1.514 | -1.726 | 0.043 | 0.035 | Cytoplasm | enzyme |
| P4HA2 | O15460 | 1.938 | 5.085 | 0.130 | 0.029 | Cytoplasm | transporter |
| PABPC1 | P11940 | -0.645 | -1.252 | 0.130 | 0.039 | Cytoplasm | translation regulator |
| PAICS | P22234 | -1.057 | -1.420 | 0.073 | 0.041 | Cytoplasm | enzyme |
| PAPOLA | P51003 | 4.844 | 0.457 | 0.033 | 0.393 | Nucleus | enzyme |
| PCNA | P12004 | -2.350 | -0.296 | 0.009 | 0.356 | Nucleus | enzyme |
| PDCD11 | Q14690 | 0.481 | 1.296 | 0.232 | 0.047 | Nucleus | other |
| PDS5B | Q9NTI5 | -0.333 | 2.043 | 0.339 | 0.029 | Nucleus | other |
| PEBP1 | P30086 | -1.575 | -1.387 | 0.022 | 0.039 | Cytoplasm | other |
| PES1 | O00541 | -0.513 | 1.533 | 0.207 | 0.032 | Nucleus | other |
| PFDN5 | Q99471 | -2.744 | -1.359 | 0.046 | 0.124 | Nucleus | transcription regulator |
| PFKM | P08237 | -1.331 | -0.261 | 0.033 | 0.357 | Cytoplasm | kinase |
| PLOD2 | O00469 | 1.333 | 3.037 | 0.023 | 0.002 | Cytoplasm | enzyme |
| PLS3 | P13797 | 1.965 | 2.980 | 0.080 | 0.030 | Cytoplasm | other |
| PPIA | P62937 | -1.069 | -1.620 | 0.047 | 0.023 | Cytoplasm | enzyme |
| PPP1R10 | Q96QC0 | -1.968 | -0.884 | 0.042 | 0.220 | Nucleus | other |
| PRDX2 | P32119 | -2.210 | -1.701 | 0.004 | 0.014 | Cytoplasm | enzyme |
| PRDX6 | P30041 | -0.779 | -2.298 | 0.164 | 0.023 | Cytoplasm | enzyme |
| PRKACA | P17612 | -1.390 | -0.618 | 0.028 | 0.171 | Cytoplasm | kinase |
| PRMT5 | O14744 | -3.785 | -0.548 | 0.022 | 0.359 | Cytoplasm | enzyme |
| PRPS1 | P60891 | 0.266 | 1.881 | 0.364 | 0.023 | Cytoplasm | kinase |
| PSMA3 | P25788 | -2.493 | 0.029 | 0.022 | 0.503 | Cytoplasm | peptidase |
| PSMA4 | P25789 | -2.366 | -0.817 | 0.003 | 0.077 | Cytoplasm | peptidase |
| PSMA5 | P28066 | -1.580 | 0.087 | 0.014 | 0.458 | Cytoplasm | peptidase |
| PSMA6 | P60900 | -2.829 | -0.932 | 0.039 | 0.270 | Cytoplasm | peptidase |
| PSMA7 | O14818 | -2.019 | -0.201 | 0.026 | 0.428 | Cytoplasm | peptidase |
| PSMB2 | P49721 | -1.290 | 0.019 | 0.037 | 0.501 | Cytoplasm | peptidase |
| PSPC1 | Q8WXF1 | -1.808 | -0.296 | 0.031 | 0.360 | Nucleus | other |
| PTN | P21246 | -3.546 | -0.053 | 0.002 | 0.485 | Extracellular Space | growth factor |
| PTPRZ1 | P23471 | -2.160 | -0.374 | 0.004 | 0.259 | Plasma Membrane | phosphatase |
| PXDN | Q92626 | 0.902 | 2.675 | 0.195 | 0.026 | Extracellular Space | enzyme |
| PYGL | P06737 | -1.250 | 1.480 | 0.044 | 0.035 | Cytoplasm | enzyme |
| RAB23 | Q9ULC3 | 1.497 | 0.297 | 0.043 | 0.394 | Cytoplasm | enzyme |
| RAB3GAP2 | Q9H2M9 | -2.144 | -0.476 | 0.015 | 0.286 | Cytoplasm | enzyme |
| RAB6A | P20340 | 1.711 | 1.190 | 0.029 | 0.078 | Cytoplasm | enzyme |
| RANBP1 | P43487 | -1.804 | -1.823 | 0.013 | 0.023 | Nucleus | other |
| RAP2B | P61225 | 2.026 | 1.072 | 0.004 | 0.041 | Plasma Membrane | enzyme |
| RBM25 | P49756 | -1.844 | -0.134 | 0.043 | 0.463 | Nucleus | other |
| RDX | P35241 | -0.622 | -1.511 | 0.146 | 0.025 | Cytoplasm | other |
| RPL13A | P40429 | 2.126 | 2.829 | 0.050 | 0.023 | Cytoplasm | other |
| RPL35A | P18077 | 0.084 | 2.479 | 0.481 | 0.007 | Cytoplasm | other |
| RPL37A | P61513 | -0.390 | 2.272 | 0.299 | 0.014 | Cytoplasm | other |
| RPLP1 | P05386 | -0.662 | -1.988 | 0.183 | 0.023 | Cytoplasm | other |
| RPS18 | P62269 | -0.097 | 1.507 | 0.469 | 0.025 | Cytoplasm | other |
| RPS26 | P62854 | 0.727 | 3.500 | 0.257 | 0.014 | Cytoplasm | other |
| RPSA | P08865 | -1.905 | 0.501 | 0.030 | 0.300 | Cytoplasm | translation regulator |
| RUVBL1 | Q9Y265 | -0.974 | 1.797 | 0.135 | 0.043 | Nucleus | transcription regulator |
| RUVBL2 | Q9Y230 | -1.397 | 1.114 | 0.042 | 0.077 | Nucleus | transcription regulator |
| SCCPDH | Q8NBX0 | 1.320 | 0.257 | 0.022 | 0.337 | Cytoplasm | other |
| SCRN1 | Q12765 | -1.129 | -2.115 | 0.069 | 0.041 | Cytoplasm | other |
| SCYL2 | Q6P3W7 | -2.771 | -0.397 | 0.043 | 0.399 | Cytoplasm | other |
| SEC23B | Q15437 | -2.213 | 0.474 | 0.031 | 0.339 | Extracellular Space | transporter |
| SELH | Q8IZQ5 | -2.776 | -0.458 | 0.034 | 0.376 | Nucleus | other |
| SEPT2 | Q15019 | -1.549 | -0.685 | 0.016 | 0.135 | Cytoplasm | enzyme |
| SEPT7 | Q16181 | -1.469 | -0.475 | 0.022 | 0.228 | Cytoplasm | other |
| SEPT9 | Q9UHD8 | -1.567 | -0.240 | 0.015 | 0.356 | Cytoplasm | enzyme |
| SET | Q01105 | -2.404 | -0.488 | 0.004 | 0.233 | Nucleus | phosphatase |
| SF1 | Q15637 | -3.533 | -0.284 | 0.047 | 0.436 | Nucleus | transcription regulator |
| SF3A1 | Q15459 | -2.050 | 0.235 | 0.013 | 0.385 | Nucleus | other |
| SF3A3 | Q12874 | -2.138 | -0.911 | 0.015 | 0.103 | Nucleus | other |
| SF3B2 | Q13435 | -1.198 | -0.785 | 0.042 | 0.114 | Nucleus | other |
| SLC1A3 | P43003 | -1.955 | -0.994 | 0.006 | 0.057 | Plasma Membrane | transporter |
| SLC25A6 | P12236 | 2.031 | 0.479 | 0.039 | 0.336 | Cytoplasm | transporter |
| SLC3A2 | P08195 | 2.606 | 1.128 | 0.005 | 0.074 | Plasma Membrane | transporter |
| SLC7A5 | Q01650 | 3.543 | 0.896 | 0.002 | 0.169 | Plasma Membrane | transporter |
| SLC9A3R1 | O14745 | 0.785 | -1.846 | 0.158 | 0.035 | Plasma Membrane | other |
| SLIRP | Q9GZT3 | -0.160 | -1.690 | 0.448 | 0.039 | Cytoplasm | other |
| SMARCA1 | P28370 | -1.262 | 0.401 | 0.046 | 0.289 | Nucleus | transcription regulator |
| SMC1A | Q14683 | -1.292 | -0.177 | 0.044 | 0.418 | Nucleus | transporter |
| SMC2 | O95347 | 0.630 | 1.921 | 0.166 | 0.015 | Nucleus | transporter |
| SMPD1 | P17405 | 0.127 | -3.051 | 0.488 | 0.037 | Cytoplasm | enzyme |
| SNRPA1 | P09661 | -1.853 | -0.997 | 0.047 | 0.174 | Nucleus | other |
| SNRPF | P62306 | -1.063 | -0.312 | 0.043 | 0.300 | Nucleus | other |
| SNX27 | Q96L92 | -3.422 | -0.707 | 0.022 | 0.293 | Cytoplasm | other |
| SOD1 | P00441 | -2.113 | -1.713 | 0.015 | 0.035 | Cytoplasm | enzyme |
| SRSF2 | Q01130 | -1.678 | 0.348 | 0.033 | 0.324 | Nucleus | transcription regulator |
| SSB | P05455 | -1.221 | -1.297 | 0.022 | 0.025 | Nucleus | enzyme |
| STMN1 | P16949 | -2.044 | -0.929 | 0.038 | 0.216 | Cytoplasm | other |
| STOML2 | Q9UJZ1 | 1.305 | -0.944 | 0.028 | 0.072 | Plasma Membrane | other |
| SUB1 | P53999 | -2.398 | 1.128 | 0.003 | 0.042 | Nucleus | transcription regulator |
| TCEB1 | Q15369 | -2.414 | -0.633 | 0.022 | 0.295 | Nucleus | transcription regulator |
| TCEB2 | Q15370 | -2.176 | 1.822 | 0.042 | 0.078 | Nucleus | transcription regulator |
| TCP1 | P17987 | -1.390 | 1.228 | 0.027 | 0.041 | Cytoplasm | other |
| TF | P02787 | -0.071 | 2.440 | 0.481 | 0.003 | Extracellular Space | transporter |
| TFCP2 | Q12800 | -4.615 | -4.549 | 0.036 | 0.041 | Nucleus | transcription regulator |
| TJP1 | Q07157 | -1.755 | -0.498 | 0.023 | 0.264 | Plasma Membrane | other |
| TMEM109 | Q9BVC6 | 1.253 | 0.151 | 0.029 | 0.416 | Cytoplasm | other |
| TMPO | P42167 | -1.194 | 0.083 | 0.029 | 0.459 | Nucleus | other |
| TNC | P24821 | -1.674 | -0.729 | 0.017 | 0.139 | Extracellular Space | other |
| TOP2A | P11388 | -1.509 | 0.540 | 0.030 | 0.234 | Nucleus | enzyme |
| TOR1AIP1 | Q5JTV8 | 1.114 | 0.528 | 0.031 | 0.169 | Nucleus | other |
| TPM4 | P67936 | -1.336 | -1.143 | 0.048 | 0.076 | Cytoplasm | other |
| TRIM25 | Q14258 | -0.840 | -1.214 | 0.077 | 0.050 | Cytoplasm | transcription regulator |
| TRRAP | Q9Y4A5 | 1.211 | 2.429 | 0.137 | 0.035 | Nucleus | transcription regulator |
| TUBB | P07437 | 1.477 | 1.640 | 0.043 | 0.038 | Cytoplasm | other |
| TUBB2A | Q13885 | -0.093 | -1.719 | 0.490 | 0.042 | Cytoplasm | other |
| TUBB2A | Q13885 | 0.896 | 3.644 | 0.135 | 0.003 | Cytoplasm | other |
| TUBB3 | Q13509 | -0.683 | -2.310 | 0.204 | 0.022 | Cytoplasm | other |
| TYW3 | Q6IPR3 | 4.498 | -0.091 | 0.038 | 0.487 | Other | other |
| UBA1 | P22314 | -1.224 | -0.866 | 0.044 | 0.101 | Cytoplasm | enzyme |
| UBE2L3 | P68036 | -2.352 | -1.107 | 0.033 | 0.157 | Nucleus | enzyme |
| UBE2M | P61081 | -1.407 | 0.220 | 0.029 | 0.386 | Cytoplasm | enzyme |
| UBE2N | P61088 | -2.250 | -0.045 | 0.018 | 0.496 | Cytoplasm | enzyme |
| UGDH | O60701 | -0.789 | 2.043 | 0.164 | 0.035 | Nucleus | enzyme |
| UGP2 | Q16851 | -2.972 | -5.663 | 0.068 | 0.034 | Cytoplasm | enzyme |
| USP39 | Q53GS9 | -1.325 | 0.559 | 0.029 | 0.188 | Nucleus | peptidase |
| VAMP7 | P51809 | -0.707 | -3.003 | 0.223 | 0.023 | Cytoplasm | transporter |
| VCL | P18206 | 0.049 | 2.321 | 0.505 | 0.011 | Plasma Membrane | enzyme |
| VPS29 | Q9UBQ0 | -0.040 | 1.439 | 0.515 | 0.039 | Cytoplasm | transporter |
| VPS33A | Q96AX1 | 5.053 | 5.117 | 0.030 | 0.037 | Cytoplasm | transporter |
| VPS45 | Q9NRW7 | -0.517 | -2.469 | 0.332 | 0.035 | Cytoplasm | transporter |
| VPS4B | O75351 | -2.764 | -1.923 | 0.028 | 0.041 | Cytoplasm | transporter |
| XPNPEP1 | Q9NQW7 | -1.915 | -0.919 | 0.044 | 0.194 | Cytoplasm | peptidase |
| XPO1 | O14980 | -1.424 | 0.546 | 0.029 | 0.216 | Nucleus | transporter |
| YWHAB | P31946 | -1.770 | -0.616 | 0.022 | 0.216 | Cytoplasm | transcription regulator |
| YWHAE | P62258 | -1.588 | -1.228 | 0.027 | 0.059 | Cytoplasm | other |
